# Integrin-beta3 clusters recruit clathrin-mediated endocytic machinery in the absence of traction force

**DOI:** 10.1038/ncomms9672

**Published:** 2015-10-28

**Authors:** Cheng-han Yu, Nisha Bte Mohd Rafiq, Fakun Cao, Yuhuan Zhou, Anitha Krishnasamy, Kabir Hassan Biswas, Andrea Ravasio, Zhongwen Chen, Yu-Hsiu Wang, Keiko Kawauchi, Gareth E. Jones, Michael P. Sheetz

**Affiliations:** 1 School of Biomedical Sciences, Li Ka Shing Faculty of Medicine, The University of Hong Kong, Hong Kong, China; 2 Mechanobiology Institute, National University of Singapore, Singapore 117411, Singapore; 3 Randall Division of Cell and Molecular Biophysics, King's College London, London SE1 1UL, UK; 4 Frontiers of Innovative Research in Science and Technology, Konan University, Kobe 650-0047, Japan; 5 Department of Biological Sciences, Columbia University, New York, New York 10027, USA

## Abstract

The turnover of integrin receptors is critical for cell migration and adhesion dynamics. Here we find that force development at integrins regulates adaptor protein recruitment and endocytosis. Using mobile RGD (Arg-Gly-Asp) ligands on supported lipid membranes (RGD membranes) and rigid RGD ligands on glass (RGD-glass), we find that matrix force-dependent integrin signals block endocytosis. Dab2, an adaptor protein of clathrin-mediated endocytosis, is not recruited to activated integrin-beta3 clusters on RGD-glass; however, it is recruited to integrin-mediated adhesions on RGD membranes. Further, when force generation is inhibited on RGD-glass, Dab2 binds to integrin-beta3 clusters. Dab2 binding to integrin-beta3 excludes other adhesion-related adaptor proteins, such as talin. The clathrin-mediated endocytic machinery combines with Dab2 to facilitate the endocytosis of RGD-integrin-beta3 clusters. From these observations, we propose that loss of traction force on ligand-bound integrin-beta3 causes recruitment of Dab2/clathrin, resulting in endocytosis of integrins.

## Abstract (web summary)

Force is known to recruit adaptor proteins to the intracellular tails of integrin extracellular matrix receptors. Here the authors show that matrix force-dependent β3 integrin signals block endocytosis by preventing the recruitment of the clathrin adaptor Dab2.

## Graphical abstract

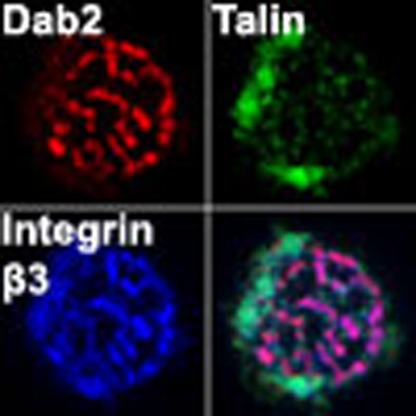

Cell migration on matrices and the organization of the extracellular matrices by cells involve mechanical as well as biochemical communication between activated integrins and the cytoskeleton^1^,^2^. An important component of that communication is the regulation of integrin dynamics, such as localization and turnover^3^. Outside-in integrin activation by extracellular ligand binding initially triggers a series of biochemical reactions, such as the recruitment of adaptor proteins and filamentous actin (F-actin) polymerization that ultimately establishes micrometre-sized adhesion clusters^4^,^5^. Force development on extracellular matrices plays a positive role in the maturation of signal transduction at integrin-mediated adhesions^6^,^7^. However, the mechanism of how matrix forces regulate integrin downregulation and endocytosis remains unclear.

We previously described how micropatterned RGD membranes allowed us to modulate the matrix force development in the extracellular microenvironment^5^,^8^,^9^. One distinct feature of mobile RGD membranes is their two-dimensional fluidity. Because of the absence of lateral force on ligands, mobile RGD membranes make it possible to investigate force-dependent and force-independent aspects of integrin signalling. More importantly, microfabricated diffusion barriers (RGD-glass) embedded in the mobile RGD membranes can serve as sites of force generation when the cell adheres and pulls on matrix ligands. Although the chemistry of ligand activation remains unchanged, micropartitioned RGD membranes enable spatial control of ligand mobility and enable the investigation of spatiotemporal signalling events at activated integrin clusters in a force-sensitive manner. In the absence of matrix force, we find that integrin-beta3 activation triggers time-dependent recruitment of different classes of adaptor proteins. Initially, classical adhesion-related molecules (such as talin) bind to activated integrin receptors^5^. If no force develops on the cell–matrix interface, then activated RGD-integrin-beta3 clusters fail to form mature adhesions and adhesion-related molecules are replaced by endocytic adaptor proteins, including Dab2.

## Results

### Dab2 binds to integrin-beta3 clusters on RGD membranes

**Figure 1:**
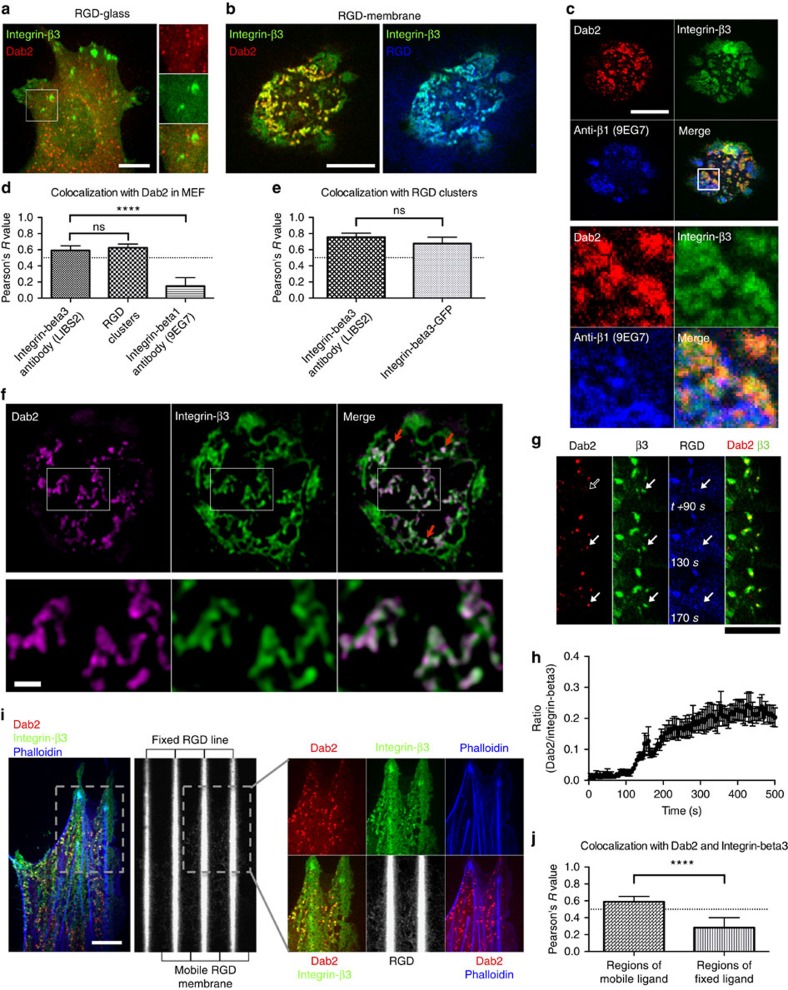
Integrin-beta3 recruits Dab2 when the cell adheres on mobile RGD membranes. (**a**) Dab2-mCherry is absent at integrin-beta3-GFP clusters when the cell is plated on RGD-glass. (**b**) Dab2-mCherry is recruited to a subpopulation of integrin-beta3-GFP clusters on RGD membranes. (**c**) RGD membranes activate both integrin-beta1 and beta3. Dab2-mCherry is only localized with integrin-beta3-GFP, not integrin-beta1 clusters (anti-beta1 clone 9EG7 staining). (**d**) Pearson's correlation analysis of Dab2 and activated integrin-beta3, RGD clusters and activated integrin-beta1. Dab2 colocalizes with integrin-beta3 and RGD clusters. (**e**) Pearson's correlation analysis of RGD and activated integrin-beta3 and integrin-beta3-GFP. RGD clusters colocalize with activated integrin-beta3 and integrin-beta3-GFP. (**f**) Two-colour structured illumination super-resolution microscopy of Dab2-mCherry and Integrin-beta3-GFP. Dab2 colocalizes with integrin-beta3 when the cell adheres on RGD membranes (red arrows). Bottom panel represents the boxed area in the upper panel. (**g**) During the initial phase of cell adhesion on RGD membranes (0–90 s), Dab2-mCherry is absent from the integrin-beta3-GFP cluster (empty white arrow). Dab2 recruitment comes after RGD-integrin-beta3 clustering (90 s and after, solid white arrows). (**h**) The area ratio between Dab2-mCherry and integrin-beta3-GFP during the initial adhesion formation. With an initial delay, Dab2 binding to integrin-beta3 increases as integrin-beta3 clusters develop. More than 1,000 RGD-integrin-beta3 clusters were analysed (*n*=6). (**i**) When the cell adheres on micropatterned RGD substrates, Dab2-mCherry is only recruited to mobile RGD-integrin-beta3 clusters. Right panel represents boxed area in the left panel. (**j**) Pearson's correlation analysis of Dab2 and integrin-beta3 colocalization on mobile or fixed RGD regions. Dab2 and integrin-beta3 are colocalized on mobile RGD regions. All data are the mean of more than three independent experiments (*n*>20 in each condition, except panel 1 h). *P* values by unpaired Kruskal–Wallis test are: ****P<0.0001 and ns *P*>0.05. Error bars represent s.d. (s.e.m. in **h**), and all samples represent biological replicates. Detailed statistical test results are available in Supplementary Table 1. Scale bar, 10 μm (except **f**) and 1 μm (**f**).

Ligand-activated integrin-beta3 cytoplasmic tails bound to a number of cytoplasmic proteins with different cellular functions, such as talin and Dab2 (refs 10, 11). Talin directly bound to integrin-beta3 at focal adhesion sites and was well documented in matrix adhesion formation^12^. Dab2 was an adaptor protein for clathrin-mediated endocytosis. However, the regulation mechanism for Dab2 binding to integrin-beta3 in the live cell has not been defined. When the distribution of Dab2 was analysed, Dab2 was not found at integrin-beta3-mediated focal adhesion sites formed on RGD-glass (Fig. 1a). However, when cells adhered on mobile RGD membranes, Dab2 was found at a subpopulation of RGD-integrin-beta3 clusters (Fig. 1b and Supplementary Fig. 1a). RGD membranes were formed with biotinylated lipids in the bilayer that were linked to biotin-RGD by fluorescent neutravidin and the diffusion coefficient of the neutravidin was ∼2 μm^2 ^s^−1^ (Supplementary Movie 1), as measured by fluorescence recovery after photobleaching^5^.

RGD ligands selectively triggered cell adhesion by activating both integrin-beta1 and beta3 (Supplementary Fig. 1b). The activation states of integrin-beta1 and beta3 were examined by conformation-specific antibodies, 9EG7 and LIBS2, respectively. When the cells adhered on RGD membranes, Dab2 was only recruited to activated integrin-beta3 sites, not to activated integrin-beta1 sites (Fig. 1c,d and Supplementary Fig. 2a,b), in agreement with previous *in vitro* biochemical studies^10^. Dab2 is often involved in the endocytosis of low-density lipoprotein receptor LRP6 (refs 13, 14). However, the majority of Dab2 was instead found to be associated with integrin-beta3 when cells adhered to RGD membranes (Supplementary Fig. 2c,d). In addition, we utilized two-colour structured illumination super-resolution microscopy and Pearson's correlation analysis to validate the colocalization of Dab2 and integrin-beta3 at the 100-nm level (Fig. 1d,f). As might be expected, Pearson's correlation analysis of RGD clusters, activated integrin-beta3 and integrin-beta3-GFP (green fluorescent protein) showed that they were colocalized (Fig. 1e). Thus, the majority of Dab2 was associated with integrin-beta3 clusters on RGD membranes, whereas Dab2 was absent at integrin-beta3 clusters on rigid RGD-glass.

To understand the dynamics of recruitment, we followed Dab2 localization during the initial stage of cell spreading on RGD membranes. RGD-integrin-beta3 clustering preceded the subsequent recruitment of Dab2 (Fig. 1g,h and Supplementary Movie 2). Over time, Dab2 was concentrated at a subpopulation of integrin-beta3 clusters (Fig. 1g). Similarly, Dab2 was also found at RGD clusters in various cell types on RGD membranes, including mouse embryonic fibroblast (MEF), REF52, H460, U2OS, HeLa and CHO cells (Supplementary Fig. 3). In addition, micropatterns of extracellular ligands by ultraviolet-based soft lithography were developed that had mobile RGD ligands in micrometre-sized domains surrounded by lines that contained immobilized RGD^15^. Dab2 was preferentially recruited to mobile RGD-integrin-beta3 clusters but not to clusters at the lines (Fig. 1i,j). Integrin-beta3 clusters that formed at immobilized RGD lines developed classic focal adhesions that were interconnected by contractile F-actin fibres. Thus, recruitment of Dab2 to activated integrin-beta3 clusters was specifically dependent on RGD lateral mobility.

### RhoA-mediated myosin contractility suppresses Dab2 binding

**Figure 2:**
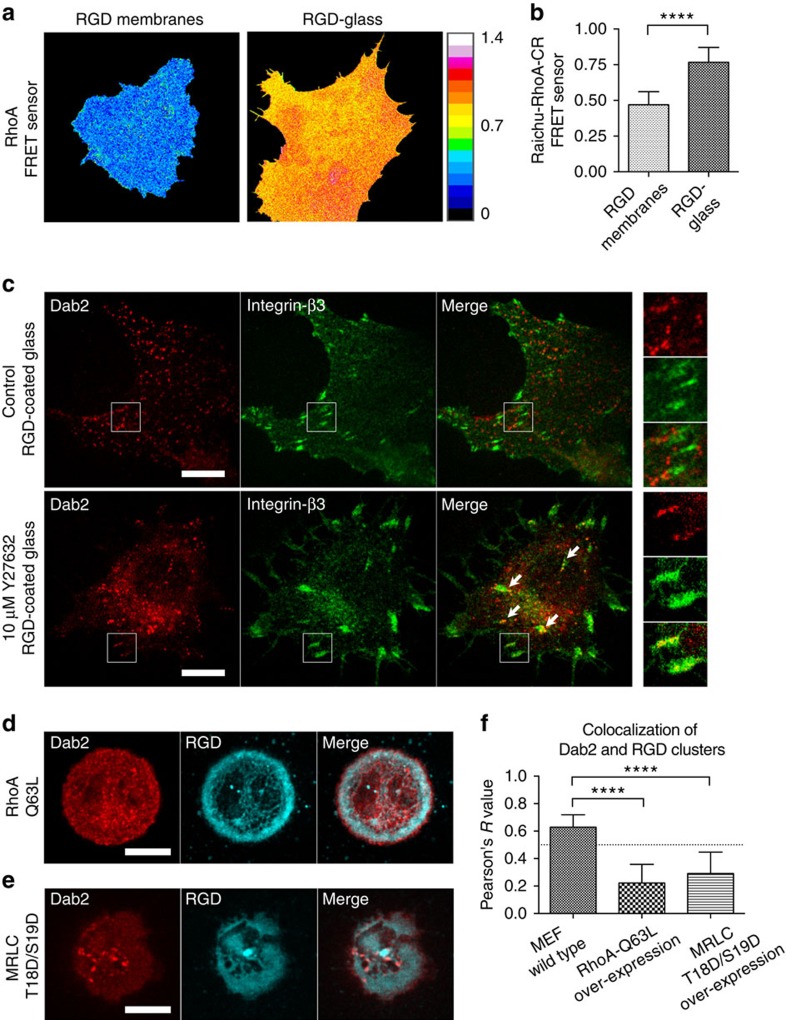
RhoA-mediated myosin-II contractility suppresses Dab2 recruitment at integrin-beta3. (**a**,**b**) FRET-based biosensor reveals that the RhoA-GTP level on RGD-glass is higher than the level on RGD membranes. (**c**) Dab2-mCherry is found at integrin-β3-GFP clusters after treatment of ROCK inhibitor (Y27632, 10 μM), even when the cell adheres on fixed RGD-coated substrate. In the control cell, Dab2 do not localize at integrin-beta3. (**d**,**e**) Overexpression of RhoA-Q63L and MRLC T18D/S19D mutants suppresses Dab2 recruitment on RGD membranes. (**f**) Pearson's correlation analysis of Dab2 and RGD clusters. With overexpression of RhoA-Q63L and MRLC T18D/S19D, Dab2 recruitment to RGD clusters is suppressed. All data are the mean of three independent experiments (*n*>25 in each condition). *P* value by unpaired Kruskal–Wallis test is: ****P<0.0001. Error bars represent s.d., and all samples represent biological replicates. Detailed statistical test results are available in Supplementary Table 1. Scale bar, 10 μm.

The distinct patterns of Dab2 recruitment at mobile but not immobile integrin clusters (Fig. 1i) further supported earlier findings that actomyosin-mediated cell–matrix contractility modulated adaptor protein associations. Immobile RGD-integrin-beta3 clusters were connected by dense F-actin stress fibres, while mobile RGD-integrin-beta3 clusters were not. We hypothesized that if actomyosin contractility and force generation on integrin-beta3 adhesions inhibited the binding of Dab2, then the inhibition of myosin contractility could facilitate Dab2 binding to RGD-integrin-beta3 clusters. Since RhoA-GTP levels are correlated with the level of contractility^16^, higher RhoA-GTP level would potentially suppress Dab2 binding to integrin-beta3. Using a FRET-based biosensor as a read-out of cellular contractility^8^,^17^, we found that the cellular RhoA-GTP level on RGD-glass was significantly higher than the level on RGD membranes (Fig. 2a,b). In the control cell on RGD-glass, Dab2 was not recruited to integrin-beta3 clusters. When myosin-II activity was suppressed by the ROCK inhibitor, Y27632 (10 μM), Dab2 was recruited to a subpopulation of integrin-beta3 clusters, even when the cell was plated on immobile RGD-glass (Fig. 2c). Likewise, when we introduced constitutively active RhoA-Q63L or overexpressed the phosphomimetic myosin regulatory light chain MRLC T18D/S19D, Dab2 recruitment was reduced on RGD membranes as measured by Pearson's correlation analysis (Fig. 2d–f). Thus, the level of RhoA activity and myosin-II force generation correlated inversely with Dab2 recruitment at integrin-beta3 clusters.

### Tyrosine mutations of integrin-beta3 alter Dab2 binding

**Figure 3:**
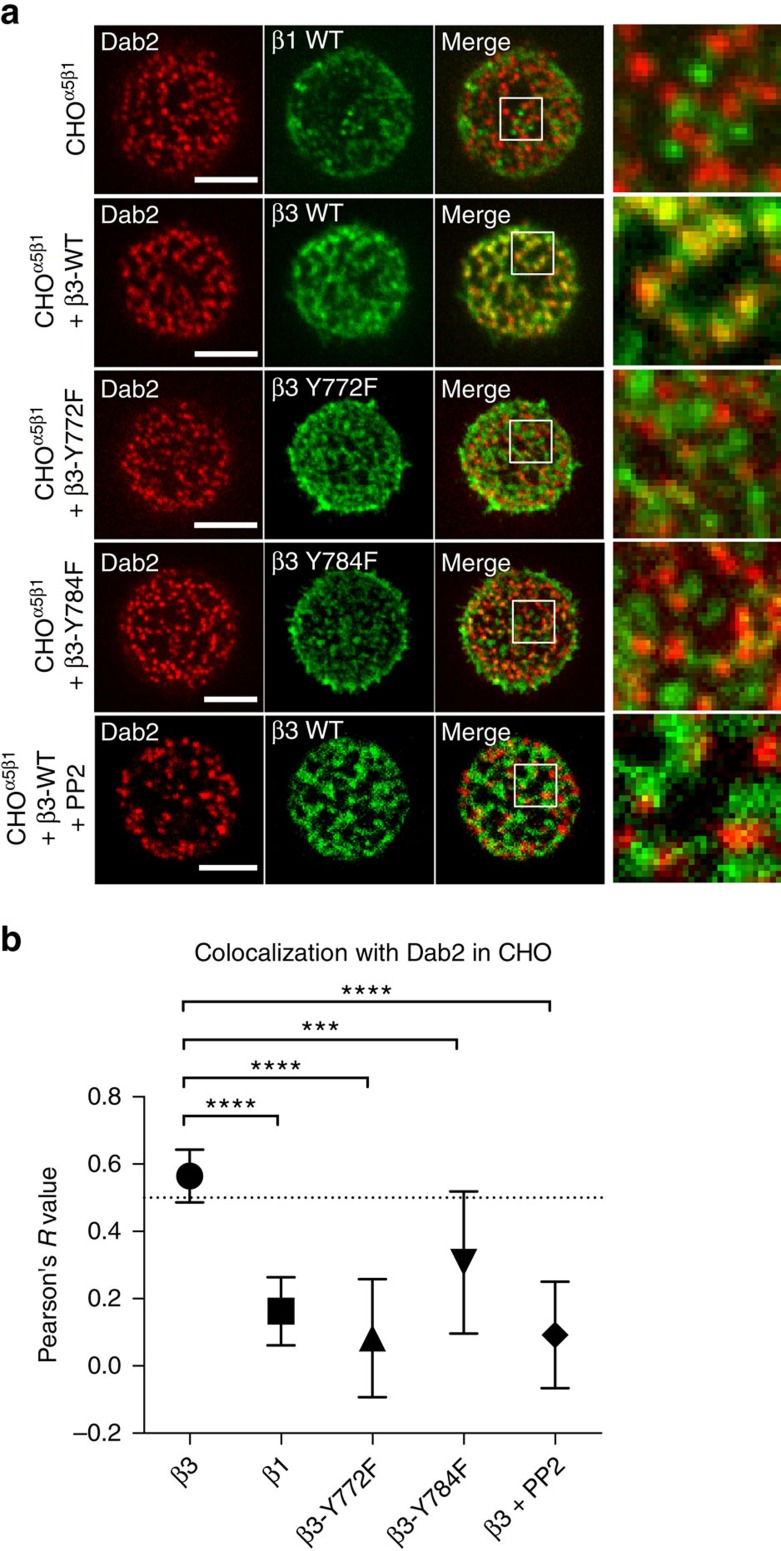
Tyrosine residues at the cytoplasmic tail of integrin-beta3 alter Dab2 binding. (**a**) Dab2-mCherry is not recruited to integrin-beta1 clusters (anti-beta1 clone 9EG7 staining) in CHO-α5β1 cells on RGD membranes. With the addition of integrin-beta3-GFP in CHO-α5β1 cells, Dab2 is then recruited at integrin-beta3-GFP clusters. Addition of integrin-beta3-Y772F-GFP or Y784F does not fully recover the Dab2 binding. Src inhibition by PP2 (10 μM) also alters Dab2 binding to integrin-beta3-GFP clusters. Also see Supplementary Fig. 4a. (**b**) Pearson's correlation analysis of Dab2 and various integrins in CHO-α5β1 cells. All data are the mean of three independent experiments (*n*>20 in each condition). *P* values by unpaired Kruskal–Wallis test are ****P<0.0001 and ***P<0.001. Error bars represent s.d., and all samples represent biological replicates. Detailed statistical test results are available in Supplementary Table 1. Scale bar, 5 μm.

Earlier studies on the *in vitro* binding of Dab2 to integrin reported that two tyrosine residues in the integrin-beta3 cytoplasmic tail regulated Dab2 binding^10^. We examined whether the mutations in mouse integrin-beta3 (Y772 and Y784) could also alter the interaction between Dab2 and integrin-beta3 in live cells on RGD membranes. Specifically, we utilized CHO cells that only expressed integrin-α5, αV and β1, but not β3 (ref. 18). When these β3-deficient CHO cells (CHO-α5β1) spread on RGD membranes, Dab2 was absent at integrin-beta1 clusters (Fig. 3a). However, after transient transfection of mouse integrin-beta3-GFP, Dab2 was recruited to integrin-beta3-GFP clusters (Fig. 3a). Point mutations of mouse integrin-beta3 cytoplasmic tyrosine residues Y772 or Y784 to phenylalanine and Src inhibition by PP2 attenuated Dab2 recruitment, as measured by Pearson's correlation analysis (Fig. 3a,b). However, a small population of Dab2 was still associated with tyrosine-mutated integrin-beta3 clusters (Supplementary Fig. 4a), in agreement with a previous study^10^. In addition, expression of a constitutively active Src kinase mutant did not promote Dab2 binding to integrin-beta3 (Supplementary Fig. 4b). Thus, it appeared that tyrosine phosphorylation of integrin-beta3 tails is not the only factor regulating Dab2 recruitment.

### Dab2 excludes classical focal adhesion proteins

**Figure 4:**
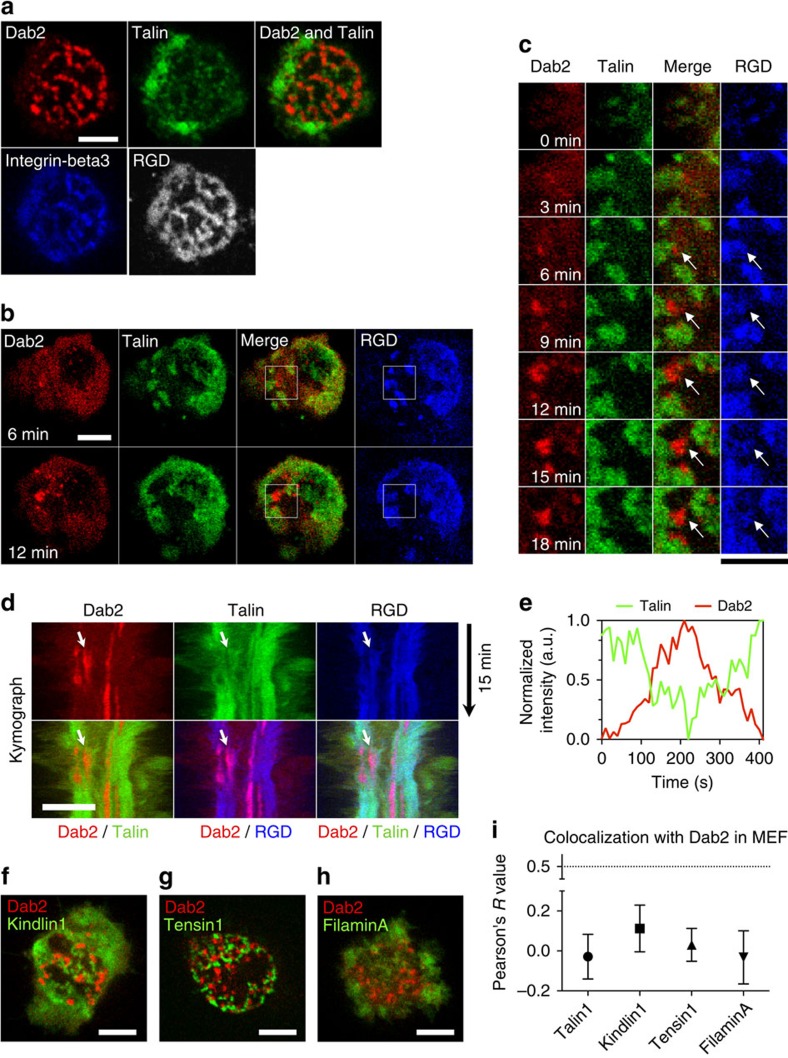
Dab2 excludes classical focal adhesion proteins at RGD-integrin-beta3 clusters. (**a**) Dab2-mCherry and GFP-talin are both recruited to RGD-integrin-beta3 clusters. Dab2 and talin are mutually excluded. (**b**,**c**) GFP-Talin is first recruited at RGD clusters, followed by Dab2-mCherry. (**c**) Time-lapsed images of the boxed area in **b**. (**d**) Representative kymograph of mutual exclusion of GFP-talin and Dab2-mCherry at RGD clusters. Dab2 replaces talin and *vice versa* (arrow). (**e**) Normalized intensity profiles of Dab2-mCherry and GFP-talin along the white arrow in panel (**d**). Dab2-mCherry and GFP-talin are inversely correlated. Also see Supplementary Fig. 5. (**f**–**h**) Dab2-mCherry is mutually excluded from Kindlin1-GFP, Tensin1-GFP and FilaminA-GFP. Also see Supplementary Movie 4. (**i**) Pearson's correlation analysis of Dab2 and adhesion-related adaptor proteins, including Talin, Kindlin1, Tensin1 and FilaminA. Dab2 and adhesion-related adhesion proteins are inversely correlated. All data are the mean of three independent experiments (*n*>20 in each condition). Error bars represent s.d., and all samples represent biological replicates. Detailed statistical results are available in Supplementary Table 1. Scale bar, 5 μm.

In the initial RGD-integrin clusters, talin was one of the key adaptor proteins that directly interacted with the integrin-beta3 cytoplasmic tail^11^,^19^ (Fig. 4b,c, 0–6 min). However, when Dab2 was bound to RGD-integrin-beta3 clusters, talin was absent (Fig. 4b,c, 6–18 min). As the level of talin at an RGD cluster dropped, the level of Dab2 increased (Fig. 4d,e; line scan along the arrow in Fig. 4d). The exchange process between Dab2 and talin was reversible (Fig. 4d,e and Supplementary Fig. 5). Thus, Dab2 and talin were always mutually exclusive at sites of recruitment to RGD-integrin-beta3 clusters (Fig. 4a and Supplementary Movie 3). To determine whether other focal adhesion adaptor proteins that bind directly to integrin-beta3, such as kindlin1, tensin1 and filaminA^20^,^21^, were similarly excluded from Dab2-integrin-beta3 clusters, we analysed the GFP chimeras of these proteins. Neither talin, kindlin1, tensin1 nor filaminA associated with Dab2 to RGD-integrin-beta3 clusters on RGD membranes, as confirmed by Pearson's correlation analysis (Fig. 4f–i and Supplementary Movie 4).

Since talin was replaced by Dab2 at early integrin-beta3 clusters over time, we tested whether stabilization of talin at adhesions would inhibit Dab2 binding. Talin recruitment to integrins was reinforced on the rigid matrices by the Rap1-RIAM activation^22^ and local enrichment of phosphatidylinositol 4,5-bisphosphate^19^,^23^. However, overexpression of constitutively active Rap1-V12 or phosphatidylinositol-4-phosphate 5-kinase type I gamma 87 (PIP5Kγ87) failed to inhibit Dab2 binding to RGD-integrin-beta3 clusters (Supplementary Fig. 6a,b,d). Further, overexpression of the calpain-cleavage-resistant talin-L432G mutant did not inhibit Dab2 binding to RGD clusters (Supplementary Fig. 6c,d). In the absence of matrix force at the forming adhesion, activation of Rap1, PIP5Kγ87 or inhibition of talin cleavage did not inhibit the substitution of talin by Dab2 when the cells adhered to fluid RGD membranes. Thus, the mobility of the integrin ligands plays the key role in modulating differential adaptor protein binding at activated integrin-beta3.

### Clathrin is recruited to integrin-beta3 by Dab2 and Numb

**Figure 5:**
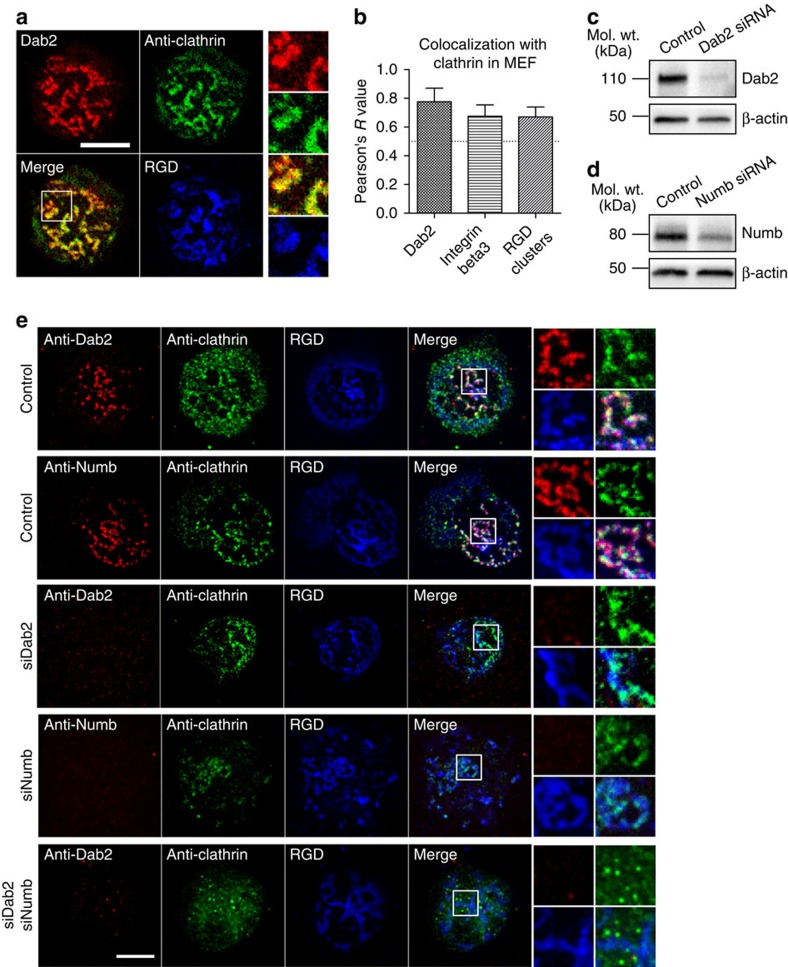
Knockdown of Dab2 and Numb suppresses clathrin localization at RGD-integrin-beta3 clusters. (**a**) Clathrin is recruited at Dab2-mCherry-positive RGD clusters. (**b**) Pearson's correlation analysis of clathrin and Dab2, integrin-beta3 and RGD clusters. Clathrin colocalizes with Dab2, integrin-beta3 and RGD clusters when cells adhere on RGD membranes. (**c**,**d**) Western blots to confirm the knockdown of Dab2 and Numb, respectively. Full blots are available in Supplementary Fig. 11. (**e**) Single knockdown of Dab2 or Numb does not abolish the clathrin recruitment at RGD-integrin clusters. Dual knockdown of both Dab2 and Numb effectively removes clathrin from RGD-integrin clusters. Also see Supplementary Fig. 8. All data are the mean of three independent experiments (*n*>20 in each condition). Error bars represent s.d., and all samples represent biological replicates. Detailed statistical results are available in Supplementary Table 1. Scale bar, 10 μm.

Because Dab2 binds to integrin-beta3 through its phosphotyrosine-binding (PTB) domain, we tested whether other PTB-domain-containing proteins^10^,^24^ such as DOK1 and LDLRAP1(ARH) bound to integrin clusters in the absence of force. DOK1-EGFP was not enriched at Dab2-bound RGD clusters, and the ARH-EGFP localization level to RGD clusters was limited (Supplementary Fig. 7a,b). However, the other PTB-domain-containing protein involved in clathrin-dependent endocytosis, Numb, was recruited to RGD clusters in a similar manner to Dab2 (Supplementary Fig. 7c,d). Both Dab2 and Numb directly bound to integrin-beta3 cytoplasmic tails *in vitro*^10^. As expected, clathrin was also recruited at Dab2-bound RGD-integrin-beta3 clusters (Fig. 5a,b).

Using an RNA interference approach, we examined whether Dab2 or Numb was required for clathrin recruitment at integrin-beta3 when cells adhered to RGD membranes. The knockdown efficiency was found to be 70–90% (Fig. 5c,d). Single knockdown of Dab2 or Numb by short interfering RNA (siRNA) did not completely abolish the clathrin colocalization with RGD clusters (Fig. 5e). However, dual knockdown of both Dab2 and Numb effectively suppressed clathrin colocalization with RGD clusters (Fig. 5e and Supplementary Fig. 8) and promoted talin colocalization with integrin-beta3 clusters (Supplementary Fig. 9). Thus, both Dab2 and Numb serve as adaptor proteins to recruit clathrin to integrin-beta3 clusters.

In addition, we tested the functional roles of Dab2 and Numb in the migration of cells seeded on compliant fibronectin-coated polydimethylsiloxane gels (CY52–276 kit, stiffness 3 kPa). Single knockdown of Dab2 or Numb caused a decrease in cell migration speed, and dual knockdown of both Dab2 and Numb resulted yet further decrease in migration speed (Supplementary Fig. 10a–c and Supplementary Movie 5).

### Endocytosis of RGD-integrin-beta3 cluster

**Figure 6:**
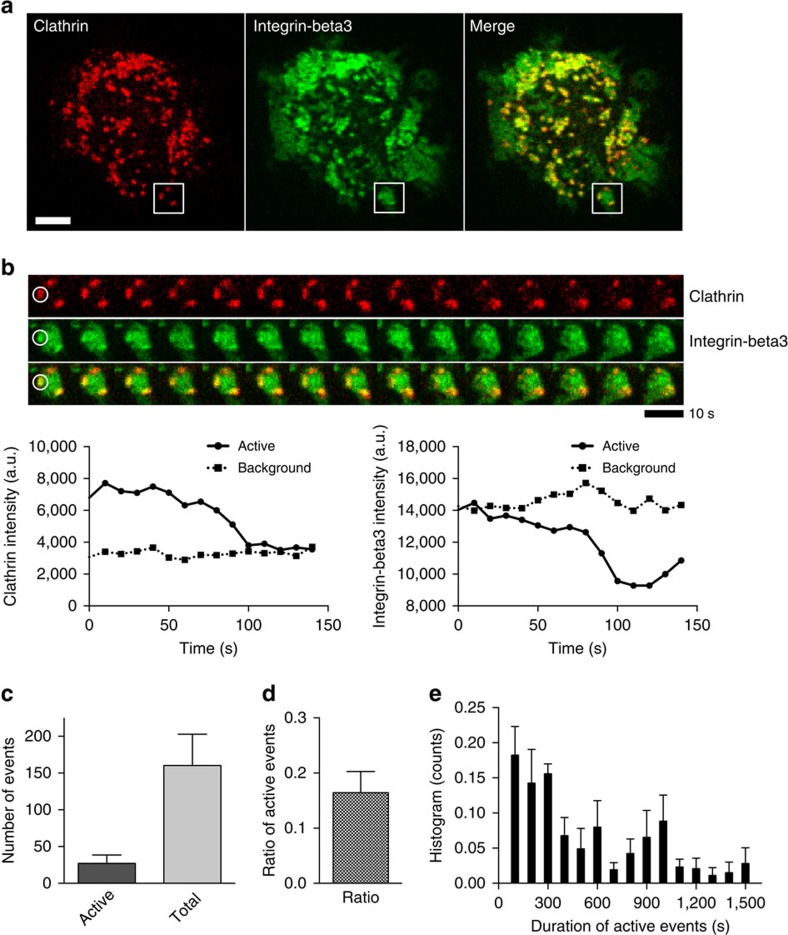
Endocytosis of RGD-integrin-beta3 cluster on RGD membranes. (**a**) Clathrin-light-chain-mCherry colocalizes with integrin-beta3-GFP on RGD membranes. (**b**) Endocytosis event of integrin-beta3. Time-lapsed images are from the boxed area in **a** and represent active clathrin puncta at integrin-beta3 clusters (circled). Intensity profiles of clathrin and integrin-beta3 puncta both decrease over time (line plot at the lower panel, active track) and indicate endocytic-active event of clathrin and integrin-beta3. Each frame is 4 × 4 μm and 10 s apart. Also see Supplementary Fig. 12 and Supplementary Movies 6 and 7. (**c**–**e**) Endocytic-active events, ratio of active versus total tracks and duration of active tracks within 30 min. All data (*n*=6, total 932 tracks analysed) are acquired from three independent experiments. Error bars represent s.d. (s.e.m. in **e**), and all samples represent biological replicates. Detailed statistical results are available in Supplementary Table 1. Scale bar, 5 μm.

These results raise the question of whether or not the binding of Dab2 causes the endocytosis of the integrin clusters and mediates adhesion turnover. Pearson's correlation analyses revealed that clathrin localized with Dab2, integrin-beta3 and RGD clusters when cells adhered to RGD membranes (Figs 5b and 6a). Other components of clathrin-mediated endocytosis^25^, such as AP2, epsin1 and dynamin2, were also found at RGD clusters (Supplementary Fig. 11a–d). To visualize active endocytic events, we monitored all Dab2 and clathrin puncta formed in images collected in a time-lapse format (total track). We then identified a subgroup of tracks (active track) that exhibited a decreased intensity profile over time in both the clathrin and integrin-beta3 channel (Fig. 6b). In these active endocytic events, intensity fluctuations of Dab2, clathrin, integrin-beta3 and RGD clusters were synchronized and diminished notably towards the end of the endocytosis process (Supplementary Movies 6 and 7). Within 30 min of observation after 3 h of adhesion formation on RGD membranes, we found that active clathrin puncta constituted ∼17% of the total (Fig. 6c,d, total 932 tracks in six cells, and Supplementary Fig. 12). The durations of active endocytosis events were widely spread with a range from 2 to 25 min (Fig. 6e).

**Figure 7:**
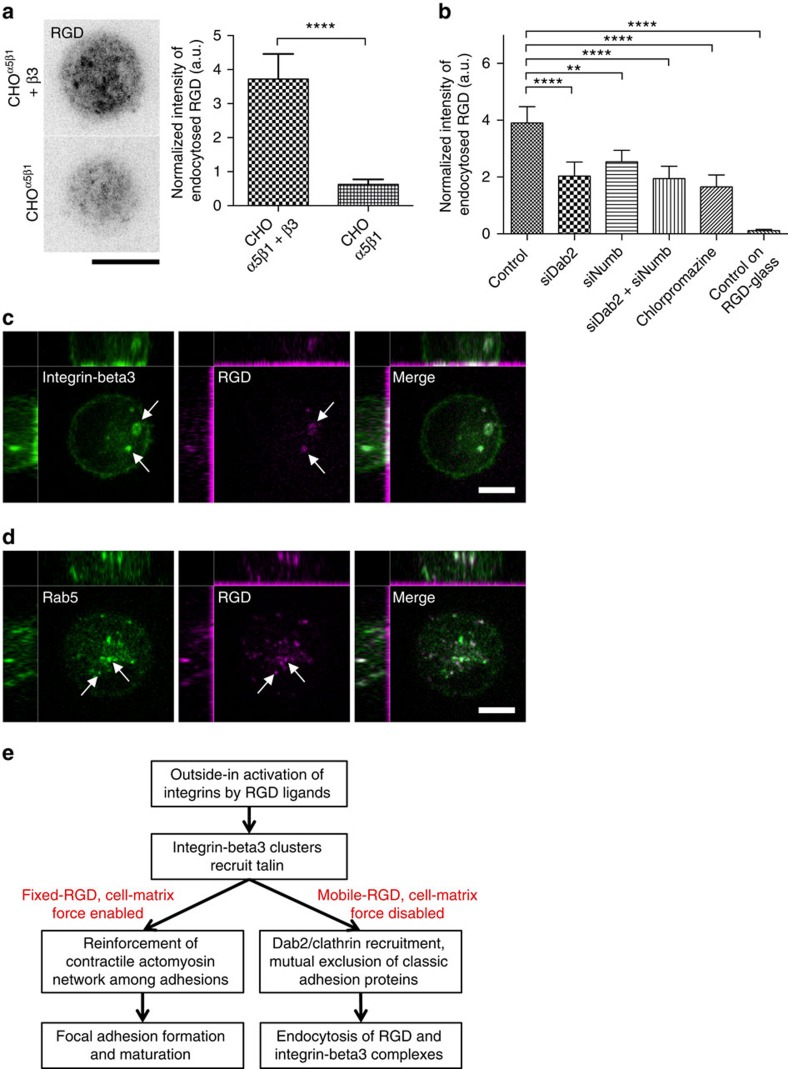
Internalization of RGD and integrin-beta3. (**a**) Endocytosis of RGD-neutravidin. Images represent inverted maximum projection of the confocal planes (1–4 μm above the substrate) in CHO-α5β1 and CHO-α5β1 cells with integrin-beta3-GFP expressed on RGD membranes. Addition of integrin-beta3 in CHO-α5β1 cells promotes endocytosis of RGD ligands. (**b**) Knockdown of Dab2 and Numb, or inhibition of clathrin coat assembly by chlorpromazine suppresses RGD-neutravidin endocytosis in MEFs on RGD membranes. Within the same time course (3 h), RGD endocytosis is not observed in MEFs that are spread on RGD-glass. Also see Supplementary Fig. 13. (**c**,**d**) Integrin-beta3-GFP and RGD-neutravidin are internalized after 3 h of cell adhesion on RGD membranes. Internalized RGD-neutravidin is sorted to mRFP-Rab5-containing vesicles. Three-dimensional confocal images are rendered with 500 nm in each z-step and total 6 μm in height. (**e**) Proposed mechanism of integrin-beta3 turnover in a cell-matrix force-dependent manner. Without cell matrix force development, activated integrin-beta3 will be internalized by clathrin-mediated endocytosis. Also see Supplementary Fig. 15. All data are the mean of three independent experiments (*n*>20 in each condition). *P* values by unpaired Kruskal–Wallis test are ****P<0.0001 and **P<0.01. Error bars represent s.d., and all samples represent biological replicates. Detailed statistical results are available in Supplementary Table 1. Scale bar, 5 μm.

To further test whether the endocytosis is specific for integrin-beta3 clusters, the amount of endocytosed RGD-neutravidin was measured in the integrin-beta3-deficient CHO-α5β1 cells using three-dimensional confocal microscopy. Total intensity of endocytosed RGD-neutravidin within the confocal volume 1–4 μm above the adhesion plane was used to quantify the endocytosis level. After 3 h of adhesion on RGD membranes, lower levels of endocytosed RGD-neutravidin were found in CHO-α5β1 cells than in the same cells when integrin-beta3-GFP was expressed (Fig. 7a). Internalization of RGD-neutravidin was suppressed by knockdown of Dab2 and Numb (Fig. 7b and Supplementary Fig. 13), as well as by the clathrin inhibitor chlorpromazine (50 μM)^26^. Other inhibitors, such as Dynasore and Pitstop2 were also examined; however, both were not compatible in our assay because of the substantial disruption of fluid bilayer structure of the entire RGD membranes. On RGD-glass, clathrin was absent at integrin-beta3 focal adhesion plaques (Supplementary Fig. 11e), and endocytosed RGD-neutravidin was not observed after 3 h of adhesion formation (Fig. 7b). In cells on RGD membranes, internalized RGD-neutravidin colocalized with integrin-beta3-GFP (Fig. 7c) and was associated with Rab5-containing vesicles (Fig. 7d). To determine whether endocytosis was limited to fibronectin-binding domains or extended to larger lipid domains, cells were bound to supported membranes functionalized with both the fibronectin fragment (FN7–10 with H10 tag) and nonspecific membrane marker, neutravidin-biotin-dextran (3 kD). Internalization of the fibronectin fragment was observed, whereas most of the dextran remained extracellular (Supplementary Fig. 14). Thus, in the absence of traction force, fibronectin (RGD)-integrin-beta3 clusters are specifically internalized via clathrin-mediated endocytosis.

## Discussion

Adhesion disassembly involves many different molecular pathways^11^, such as paxillin phosphorylation^27^ and the removal of integrin receptors from the plasma membrane by endocytosis. In particular, endocytosis of integrin receptors plays an important role in adhesion turnover, cell migration and cancer metastasis^28^. Here we demonstrate that the loss of physical forces on ligand-bound integrins can switch downstream signalling of integrin-beta3 activation from classic focal adhesion formation to a pathway of Dab2 binding and clathrin-mediated endocytosis (Fig. 7e and Supplementary Fig. 10). While integrin-beta3 is activated via the same ligand chemistry, RGD-glass allows integrin-beta3 adhesion plaque assembly and activates RhoA-mediated actomyosin contractility. Dab2 and clathrin are absent at integrin-beta3 focal adhesion sites. However, the lateral mobility of the RGD ligands on the supported membranes still triggers integrin-beta3 activation but frustrates the development of force from actomyosin contractility^8^. On RGD membranes, we find that Dab2 and other clathrin-related adaptor proteins are subsequently enriched at a subpopulation of integrin-beta3 clusters, with concomitant loss of talin and other adhesion-related adaptor proteins (Fig. 4).

In agreement with a prior study^10^, the phosphorylation of integrin-beta3 tails alters Dab2 binding (Fig. 3). However, a recent report based on mass spectrometry has confirmed the phosphorylation of integrin-beta3 tyrosines, Y772 and Y784, when cells adhere to a fixed fibronectin-coated substrate^29^. Thus, the absence of Dab2 at integrin-beta3 clusters on a fixed matrix is not caused by a deficiency in tyrosine phosphorylation. In addition, Src is one of the tyrosine kinases that phosphorylates integrin receptors; however, expression of constitutively active Src kinase (Src-Y527F) does not promote Dab2 binding to integrin-beta3 on fixed substrates (Supplementary Fig. 4b). We also find that integrin-beta3-Y772F and Y784F mutants still associate with a small population of Dab2 on RGD membranes (Supplementary Fig. 4a). The lack of tyrosine phosphorylation on integrin-beta3 fails to completely block Dab2 binding. Therefore, we believe that the differential recruitment of Dab2 to integrin clusters on RGD membranes versus RGD-glass is not entirely regulated by tyrosine phosphorylation of integrin-beta3.

Our observations indicate that a decrease in myosin-II contractility by Y27632 and an increase in extracellular ligand mobility can both promote Dab2 binding to integrin-beta3. Likewise, increases in myosin-II contractility and immobilization of extracellular ligand both suppress Dab2 binding to integrin-beta3 clusters. In addition, a micropatterned substrate with both mobile and fixed ligands directly reveals that Dab2 preferentially binds to integrin-beta3 in regions containing mobile ligands (Fig. 1i,j). Thus, we suggest that the development of actomyosin contractility inhibits Dab2 binding to integrin-beta3, and the loss of cell–matrix force development is one of the key mechanisms activating Dab2 binding to integrin-beta3 clusters.

Previously, protein kinase C (PKC) and atypical PKC-dependent pathways of Dab2 and Numb binding to integrin receptors have been investigated^30^,^31^. On RGD membranes, Dab2 and Numb bind to integrin-beta3 in a similar manner and both serve as adaptor proteins to facilitate clathrin-dependent endocytosis^14^. Knockdown of both Dab2 and Numb effectively blocks clathrin localization to RGD clusters and suppresses the endocytosis of RGD-neutravidin (Figs 5e and 7b). Similar decreases in RGD endocytosis in single and double knockdown cells may suggest a potential collaborative interaction of Dab2 and Numb, which remains to be further investigated. Endocytosis of RGD-neutravidin is a clathrin-dependent process, as chlorpromazine effectively suppresses the level of endocytosis (Fig. 7b). In contrast to the fast endocytosis of non-adhesion-related membrane receptors, ligand-bound integrin receptors physically form strong adhesion plaques and are endocytosed at a slow rate (Fig. 6e), when compared with the endocytosis rate of transferrin receptor^32^. While Dab2/clathrin-bound puncta on integrin-RGD clusters are larger than typical diffraction-limited clathrin-coated structures, these larger puncta remain endocytically active^32^,^33^. Overall, ∼17% of Dab2/clathrin-bound puncta were endocytically active within 30 min of observation when observed after 3 h of adhesion formation on RGD membranes.

Internalized integrin-beta3 puncta that colocalize with RGD ligands indicate that endocytosis occurs at the basal surface of the cell over the RGD membranes, and these are found at Rab5-containing early endosomes (Fig. 7c,d). During the endocytic events (Fig. 6b), the slow and persistent intensity drop in both clathrin and integrin-beta3 channels may involve other rate-limiting steps to facilitate endocytosis processes. Nevertheless, the force required to extract lipid-bound RGD-neutravidin is relatively low and previously measured as 3.4–6.1 pN (ref. 34). The long lifetime of Dab2/clathrin puncta and the slower pace of integrin-beta3 endocytosis may reflect the generally slow turnover rate of the ligand-bound integrins. Likewise, slower endocytosis of integrin and other ligand-bound membrane receptors has also been documented elsewhere^35^,^36^,^37^.

The contractility-dependent binding of Dab2 is specific for Integrin-beta3. Dab2 and clathrin are not bound to activated integrin-beta1 clusters on RGD membranes. This indicates that the turnover of integrin-beta1 may be regulated by a different pathway^38^, and previous studies indicate that beta1 turnover is insensitive to cell matrix forces^39^. In the general context of matrix adhesion development, early adhesions are typically mediated by integrin-beta3 and then often undergo a transition to integrin-beta1-based adhesions. This transition from beta3 to beta1 integrin is correlated with actomyosin contractility^40^. To further examine the level of integrin internalization, integrins tagged with a photo-activatable fluorescent protein can be utilized to monitor the spatiotemporal reorganization of integrin receptors in a time-dependent manner^41^. A point to note for consideration and future investigation concerns the effect of matrix mechanical properties in cancer cell invasion^42^. In cases where matrix metalloproteinase activities are upregulated in metastatic cancer cells, the matrix components are locally degraded and thus become effectively mobile^43^. Thus, proteolytically released matrix will physically resemble the mobile ligands in RGD membranes used here and would activate endocytosis of integrins that would further contribute to tissue disorganization^44^.

Despite identical ligand chemistry, force development at the cell–matrix interface (RGD-glass) reinforces talin association and inhibits Dab2 binding to integrin-beta3. However, when matrix force development is impaired (RGD membranes), Dab2 association with integrin-beta3 is enabled. The mutually exclusive pattern of Dab2 and talin binding to integrin-beta3 clusters on RGD membranes confirms that the binding domains overlap in the integrin-beta3 cytoplasmic tail. Remarkably, RGD membranes allow us to directly visualize the spatiotemporal association of Dab2 and endocytosis of integrin-beta3. While normal matrix proteins might not be as mobile as the RGD membranes, the proteolytically degraded matrices will be mobile, and our micropatterned RGD membrane system provides a demonstration that differential recruitment of Dab2 to mobile sites of integrin clusters can occur at a subcellular level.

In summary, we propose that mobile matrix ligands redirect conventional integrin signalling, recruit Dab2 and clathrin and ultimately trigger endocytosis of integrin-beta3. While only 17% of Dab2-bound integrin-beta3 puncta go through the endocytic pathway within the 30-min time frame we utilize, the repeated binding of Dab2 will assure eventual endocytosis. Because Dab2 and talin are mutually exclusive at integrin clusters, they provide a digital switch to re-programme integrin-related adhesion signalling as matrix compliance or force generation changes. Our results of adhesion transformation highlight a critical role of microenvironment sensing that involves both biochemical and mechanical steps to regulate signal transduction.

## Methods

### Cell culture

RPTPα+/+ MEFs^45^, REF52, H460, U2OS and HeLa cells were used in this study. CHO-α5β1 cells were generous gifts from Dr Siobhan A. Corbett^18^. Unless otherwise stated, MEFs were utilized in the experiments. Cell lines were grown in DMEM medium supplemented with 10% (v/v) heat-inactivated fetal bovine serum, 100 U ml^−1^ penicillin, 100 μg ml^−1^ streptomycin and 20 mM HEPES in 37 °C incubators with 5% CO_2_. CHO-α5β1 cells were grown in the similar medium with Antimycotic antibiotics, 1% nonessential amino acids and G418 (250 μg ml^−1^). DMEM media, heat-inactivated fetal bovine serum, penicillin, streptomycin, Antimycotic, nonessential amino acids, HEPES, TrypLE Express (trypsin-like protease) and Neon electroporation kits were purchased from Life Technologies (Grand Island, NY, USA).

### DNA constructs and point mutants

The plasmids with fluorescent fusion protein were transiently transfected via the Neon electroporation system, including Integrin-beta3-GFP^46^, GFP-talin^12^, Dab2-mCherry and Dab2-GFP^24^, Kindlin1-GFP^47^, Tensin1-GFP^48^, FilaminA-GFP^49^, EGFP-RhoA-Q63L (Addgene #12968), pEGFP-MRLC1-T18D-S19D phosphomimetics mutant (Addgene #35682), mCherry-Clathrin light chain (Addgene #27680), Clathrin-LCa-EYFP (Addgene #21741), mRFP-Rab5 (Addgene #14437), pCS2 CA-LRP6-GFP (Addgene #29682), LDLRAP1-EGFP^24^, GFP-Rap1-V12 (ref. 50), GFP-PIP5Kγ87 (Addgene #22300), GFP-Talin-L432G (Addgene #26725), AP2u2-mCherry (Addgene #27672), Epsin1-ECFP (Addgene #21066), Dynamin2-GFP (Addgene #34686), Src-Y527F^8^ and Raichu-RhoA biosensor^17^. Integrin-beta3-EBFP2 was subcloned by swapping GFP of integrin-beta3-GFP with EBFP2 (Addgene #14893). DOK1-EGFP and Numb-EGFP were subcloned with Gateway donor cDNA (DNASU #HsCD00512351 and HsCD00506082, respectively) and destination vector pDest-eGFP-N1 (Addgene #31796). Unless otherwise stated, 1 μg of plasmid was used to achieve low expression in each transfection.

The Stratagene QuikChange site-directed mutagenesis kit was used to generate point mutation at two tyrosine sites in integrin cytoplasmic tail. Mouse integrin-beta3-GFP Y772F point mutant was generated by forward primer 5′-CAAACAACCCGCTGTTTAAAGAGGCCACCTCCA-3′ and reverse primer 5′-TGGAGGTGGCCTCTTTAAACAGCGGGTTGTTTG-3′. Mouse integrin-beta3-GFP Y784F point mutant was generated by forward primer 5′-TTCACCAATATCACCGAACGGGGGACTTCACC-3′ and reverse primer 5′-GGTGAAGTCCCCCGTTCGGTGATATTGGTGAA-3′.

### siRNA

RNAi knockdown of Dab2 and Numb in RPTPalpha MEF was performed using ON-TARGETplus Mouse Dab2 and Numb siRNA purchased from Dharmacon (Lafayette, CO, USA). siRNA pools were transfected by electroporation, either separately or in combination, at a final concentration of 50 nM and a cell density of 10^7^ cells ml^−1^ using the manufacturer's protocol. Efficiency of RNAi knockdown was analysed after 36 h of transfection by western blot analysis using antibodies specific to Dab2 and Numb. Rabbit monoclonal antibodies for Dab2 and Numb (#12906 and #2756, respectively) were purchased from Cell Signaling Technology (Danvers, MA, USA) and were used at a dilution of 1:1,000 for immunodetection. β-actin was used as an internal loading control and was detected using mouse monoclonal anti-β-actin antibody purchased from Abcam (Cambridge, MA, USA). The knockdown efficiency was found to be 70–90%. The full blots are available in Supplementary Fig. 16.

### RGD-supported lipid bilayer membranes

Detailed preparation methods were previously described^5^. 1,2-dipalmitoyl-sn-glycero-3-phosphoethanolamine-N-(cap biotinyl) (16:0 biotinyl-Cap-PE), 1,2-dioleoyl-sn-glycero-3-[(N-(5-amino-1-carboxypentyl)iminodiacetic acid)succinyl] nickel salt (Ni-NTA-DOGS) and 1,2-dioleoyl-sn-glycero-3-phosphocholine were purchased from Avanti Polar Lipids (Alabaster, AL, USA). Final concentration of 0.06–0.12% of biotinyl-Cap-PE or 4% of Ni-NTA-DOGS was used in the supported lipid membranes. Neutravidin with Cascade Blue or DyLight 680 was purchased from Life Technologies or Thermo Fisher Scientific Inc. (Rockford, IL, USA), respectively. Biotinylated RGD, cyclo [Arg-Gly-Asp-D-Phe-Lys(Biotin-PEG-PEG)], RGE and RAD peptides were purchased from Peptides International Inc.(Louisville, KY, USA). Fibronectin repeat III 7th to 10th domain monomer with 10 histidines (FN7–10 H10) was purified using the method previously described^51^, and then labelled with CF680R maleimide. The lipid membrane-coated glass substrate was then assembled with an Attofluor cell chamber (Life Technologies) or Chamlide magnetic chamber (Live Cell Instrument, Seoul, Korea) within the water bath at room temperature. After assembly, ligand-coated supported lipid membranes in the chamber were always kept under aqueous conditions.

Micropatterned RGD lines were achieved by direct deep-ultraviolet patterning^15^. Biotin-conjugated silane (PG2-BNSL-2k, Nanocs Inc., New York, NY, USA) or biotinylated bovine serum albumin was first coated and immobilized on cover glass. A quartz photomask with microfabricated chromium patterns was attached on coated cover glass. Deep-ultraviolet light was used to locally remove/degrade biotinylated glass coating through the photomask (typically 5-min exposure). Biotinylated lipid membranes were then backfilled to cover the area where the biotinylated coating was removed. Surface densities of available biotins over both mobile and immobile areas remained equivalent (0.06–0.12%). Complementary micropatterned surfaces with mobile (biotinylated supported lipid membranes) versus immobile (biotinylated glass coating) ligands were established. Further functionalization of RGD was followed by the method described above.

### Elastic substrate

For the cell migration assays, a soft polydimethylsiloxane (3 kPa) gel was prepared by 1:1 mixture of the CY52–276A/B kit (Dow Corning), as previously described^52^. Briefly, CY52–276A and CY52–276B were mixed in a 1:1 ratio, and 100 μl of silicon mixture was deposited on a glass-bottom Petri dish, spin-coated at 500 r.p.m. for 45 s and cured at 80 °C for 2 h. Cured silicon substrates were then treated with oxygen plasma for 15 s, incubated with fibronectin at the concentration of 25 μg ml^−1^ for 30 min at 37 °C and then washed three times with 1 × PBS to remove unbound fibronectin. Cells were stained with 5 μM far-red DNA dye DRAQ5 (Life Technologies) 30 h after the siRNA transfection and seeded at low confluency to minimize cell–cell contacts during migration. Cells were allowed to adhere and spread on the substrate for 6 h before imaging.

### Immunofluorescence and inhibition chemicals

Anti-integrin-β3 monoclonal antibody (LIBS2) was purchased from EMD Millipore (Billerica, MA, USA). Anti-integrin-β1 monoclonal antibody (#550531) and Anti-Clathrin heavy chain monoclonal antibody (#610499) were purchased from BD Biosciences (San Jose, CA, USA). Anti-Dab2 monoclonal antibody (D709T) was purchased from Cell Signaling (Boston, MA, USA). Anti-Numb polyclonal antibody (#ab14140) was purchased from Abcam. For fixed cell experiments, cells were fixed with 4% flesh-prepared paraformaldehyde, permeabilized with 0.05% Triton X and blocked with 5% casein or BSA overnight. Primary antibodies were used in 1:200 dilution, followed by secondary antibodies in 1:1,000 dilution. Micro-ruby dextran (3 kD, with both tetramethylrhodamine and biotin) with a biotin tag was purchased from Life Technologies. CF680R maleimide and phalloidin labelled with CF405 or CF680R dye were purchased from Biotium (Hayward, CA, USA). ROCK inhibitor Y27632, Src inhibitor PP2 and chlorpromazine were purchased from Sigma-Aldrich (St Louis, MO, USA). Chemicals were first kept as a stock concentration 1,000 times higher than the final concentration. Before applying to cells, chemicals were diluted 1,000 times into DMEM media.

### Microscopy

Fluorescent images were taken with an inverted spinning-disk confocal microscope (PerkinElmer UltraVIEW VoX), with × 100 oil immersion lens (1.40 numerical aperture, UPlanSApo × 100, Olympus) and cooled EMCCD camera (C9100-13, Hamamatsu Photonics). An environmental chamber (37 °C and 5% CO_2_) was attached to the microscope body for long-term time-lapse imaging. Two-colour structured illumination super-resolution images were taken with a Nikon N-SIM inverted microscope, with × 100 oil immersion lens (1.49 numerical aperture, CFI Apochromat TIRF × 100, Nikon) and cooled EMCCD camera (DU-897, Andor). Fifteen images (three directions and five phases) were taken at each position and then reconstructed using the NIS-Elements software. FRET images of RhoA-Raichu-CR biosensor were acquired using 488-nm laser excitation and 580–650-nm emission filter. Green channel was used as the reference baseline and was monitored by 488-nm laser excitation with 500–555-nm emission filter. Cell migration was visualized with both phase contrast (cell morphology) and far-red fluorescence (cell nucleus) channels on a fully automated Olympus IX81 inverted microscope with a × 10 air objective lens.

### Endocytosis assay

Cells were first re-plated on to RGD membranes to allow cell attachment and new adhesion formation. Samples with RGD membranes were maintained in 37 °C to prevent artificial aggregation of RGD-neutravidin. Examination of the endocytic compartment started after 2 h of initial adhesion, and cells were fixed in 3 h. Under identical acquisition parameters, endocytosed RGD-neutravidin was visualized using confocal microscopy with 500 nm per section and 4 μm depth in total. In the first hour, the RGD signal in the endocytic compartment was often too low to be observed. Three-dimensional views of the confocal images were rendered using Volocity (PerkinElmer Inc., Waltham, MA, USA). Z-stack images (from 1 to 4 μm) were added up and used to compare the level of endocytosis. Regions of interest within the cell and without the cell were used to quantify the internalized RGD intensity and background intensity, respectively.

### Data analyses

Colocalization analysis was performed with Coloc2 in ImageJ (NIH, Bethesda, MD, USA). Region of interest within the cell adhesion footprint was selected, and Pearson's correlation coefficients (*R*) were calculated between two channels without threshold. Time-lapsed intensity measurement was performed using Imaris (Bitplane AG, Zurich, Switzerland). For the RhoA analysis, the FRET ratio was calculated after background subtraction in both channels^8^,^53^. Three or more independent experiments were repeatedly performed under each condition, and all samples represented biological replicates. Statistical bar graphs with the mean and error bars were plotted using Prism (GraphPad Software Inc., La Jolla, CA, USA). Statistical significance analysis was performed using Prism (Kruskal–Wallis test), and *P* values were calculated (**P<0.01; ***P<0.001; and ****P<0.0001). Detailed statistical test results are available in Supplementary Table 1.

## Additional information

**How to cite this article:** Yu, C.-H. *et al*. Integrin-beta3 clusters recruit clathrin-mediated endocytic machinery in the absence of traction force. *Nat. Commun.* 6:8672 doi: 10.1038/ncomms9672 (2015).

## Supplementary Material

Supplementary Information Supplementary Figures 1-16 and Supplementary Table 1

Supplementary Movie 1 Lateral fluidity of RGD ligands on supported lipid membranes

Supplementary Movie 2 Recruitment of Dab2 at integrin-beta3 cluster

Supplementary Movie 3 Dab2 and talin mutually exclude each other at RGD clusters

Supplementary Movie 4 Dab2-mCherry is mutually excluded from Kindlin1-GFP, Tensin1-GFP, and FilaminA-GFP

Supplementary Movie 5 Migration tracks of control, siDab2, siNumb, and siDab2+siNumb cells on fibronectin-coated PDMS gel (3kPa)

Supplementary Movie 6 Endocytosis of integrin-beta3-GFP

Supplementary Movie 7 Endocytosis of RGD ligand
