# Supplementary material for: Integrin-beta3 clusters recruit clathrin-mediated endocytic machinery in the absence of traction force
Source: Nat Commun. 2015 Oct 28;6:8672. doi: 10.1038/ncomms9672 (PMC4846324; doi:10.1038/ncomms9672)
Supplement: Supplementary Information — Supplementary Figures 1-16 and Supplementary Table 1 [file ncomms9672-s1.pdf]

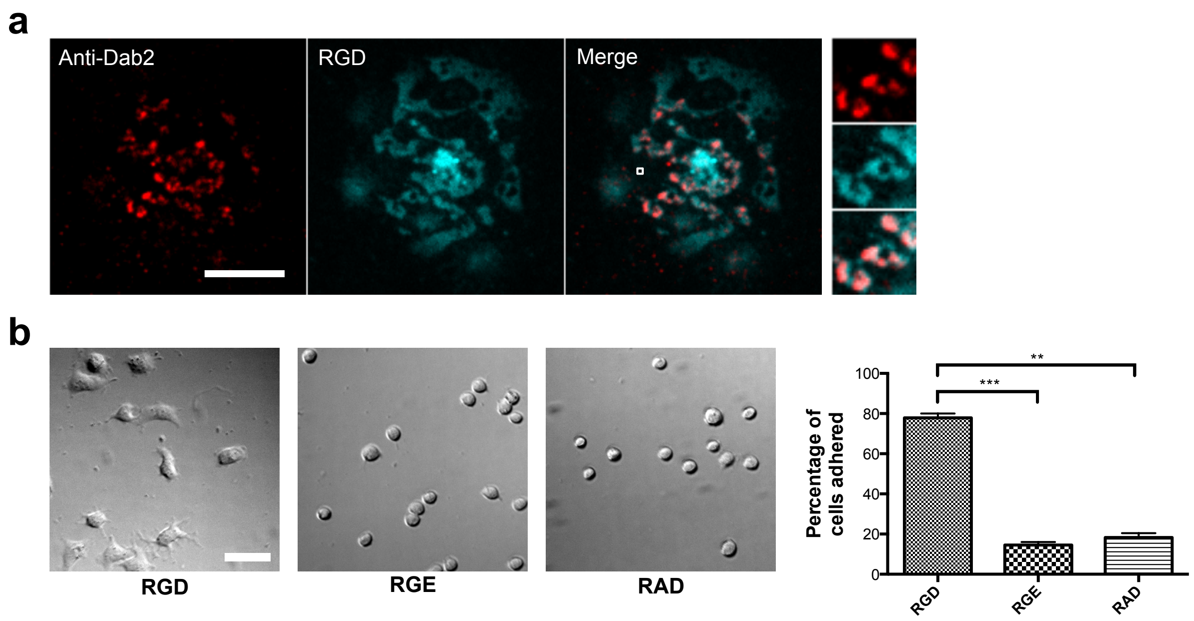

Supplementary Figure 1. Dab2 are localized at RGD clusters. (a) Endogenous Dab2 is localized at RGD clusters. (b) RGD, but not RGE nor RAD peptides can trigger cell adhesion. Peptides are pre-coated on the coverglass. After 2hr, majority of MEFs form spread morphology on RGD-coated glass. On RGE and RAD coated glass, majority of MEFs do not spread. All data are the mean of three independent experiments (n > 20 in each condition). P values by unpaired Kruskal–Wallis test are: *** P < 0.001, and ** P < 0.01. Error bars represent s.d., and all samples represent biological replicates. Detailed statistical test results are available in Supplementary Table 1. Scale bar represents 10μm in panel (a) or 100μm in panel (b).

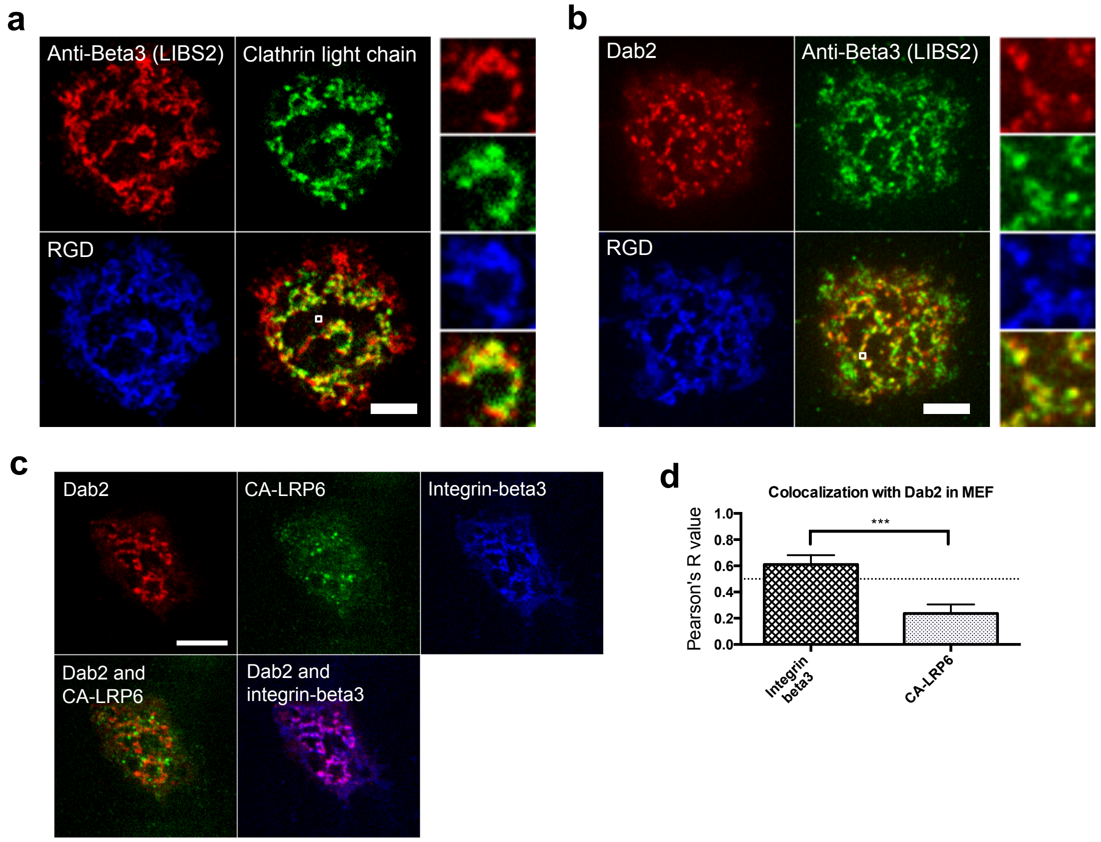

Supplementary Figure 2. Clathrin and Dab2 are localized at activated RGD-integrin-beta3 clusters. (a) Conformation-specific antibody LIBS2 confirms that integrin-beta3 is activated and localizes with RGD clusters. Clathrin light chain-EYFP also localizes with activated integrin-beta3. (b) Dab2-mCherry localizes with activated integrin-beta3 clusters. (c) When adhered on RGD-membranes, majority of Dab2-mCherry are not localized to CA-LRP6-GFP (constitutively-active LDL-receptor related protein 6, lacking its extracellular domain). Dab2-mCherry localizes with integrin-beta3-BFP2 clusters. (d) Pearson's correlation analysis of Dab2/integrin-beta3 and Dab2/CA-LRP6. All data are the mean of three independent experiments (n > 20 in each condition). P value by unpaired Kruskal–Wallis test is: *** $P < 0.001$. Error bars represent s.d., and all samples represent biological replicates. Detailed statistical test results are available in Supplementary Table 1. Scale bar represents 10μm.

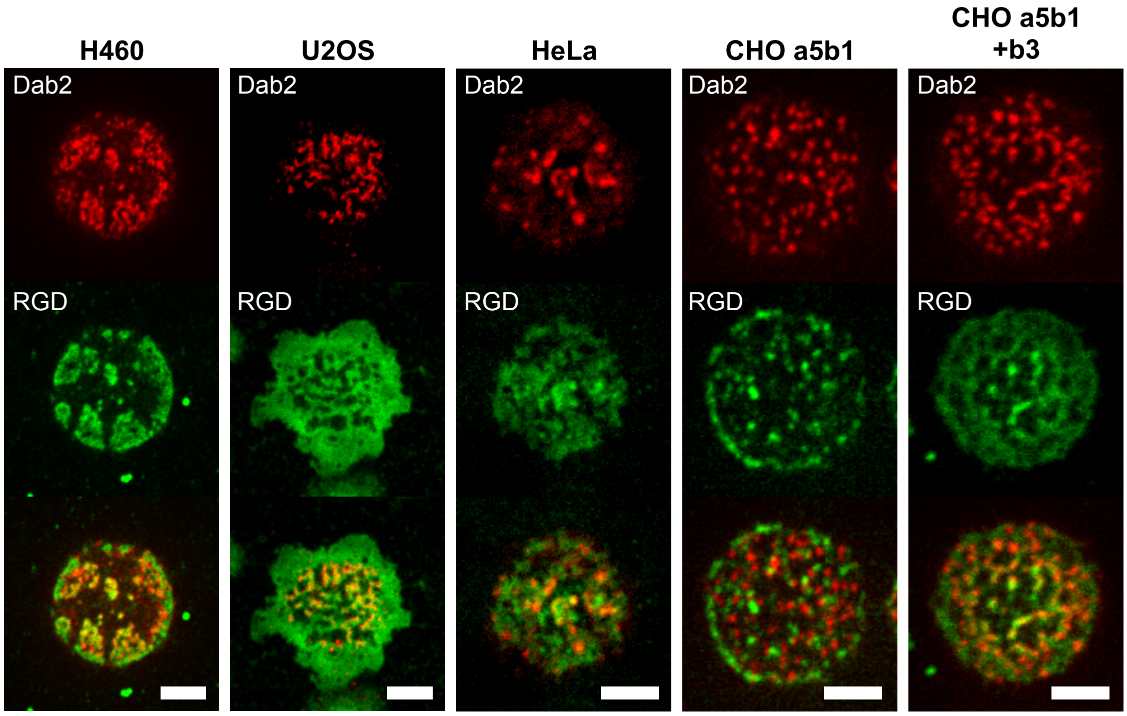

Supplementary Figure 3. Dab2 are localized at RGD-integrin clusters in various cell types. Dab2-mCherry is found at RGD clusters in various cell types on RGD-membranes, including H460, U2OS, HeLa, and CHO cells. However, Dab2-mCherry is absent from RGD clusters in the CHO cells lacking integrin-beta3. Scale bar 5μm.

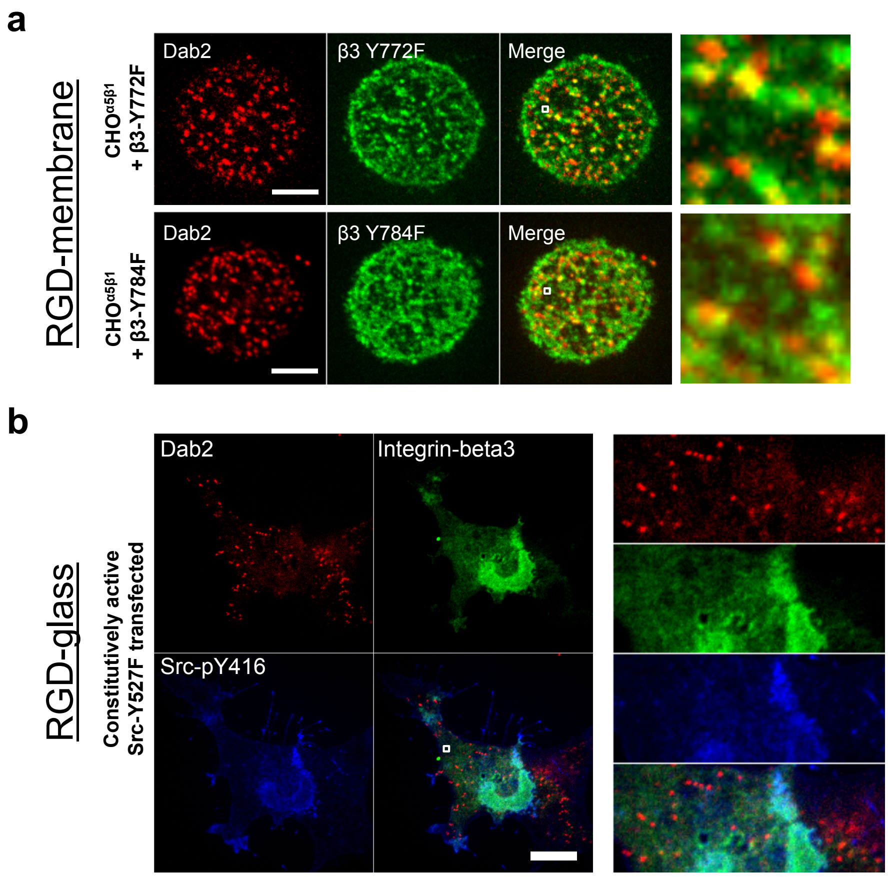

Supplementary Figure 4. Dab2 binding is not solely dependent on Integrin-beta3 tyrosine phosphorylation. (a) Tyrosine to phenylalanine mutation of integrin-beta3 fails to completely block Dab2 binding. Co-localization of Dab2-mCherry and integrin-beta3-Y772F-GFP or Y784F can still be observed on the RGD-membranes. Scale bar represents 10µm. (b) Introduction of constitutive active Src kinase mutant does not promote Dab2 binding to integrin-beta3 on RGD-glass. Src activity, visualized by Src-pY416 staining is mainly at integrin-beta3 clusters that do not associate with Dab2. Scale bar represents 5µm.

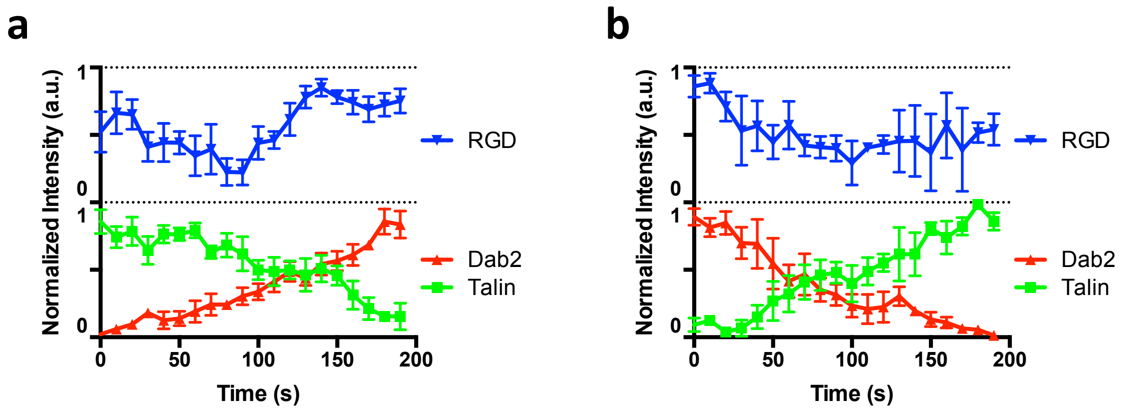

Supplementary Figure 5. Inversely correlated recruitment of Dab2 and talin. (a) As Dab2 is recruited over time, talin begins to dissociate. (b) When Dab2 begins to dissociate, talin recruitment is increased. The intensity fluctuation of RGD suggested that integrin-beta3 were dynamically reorganized during the Dab2/talin exchange. Error bars represent s.e.m (n=6).

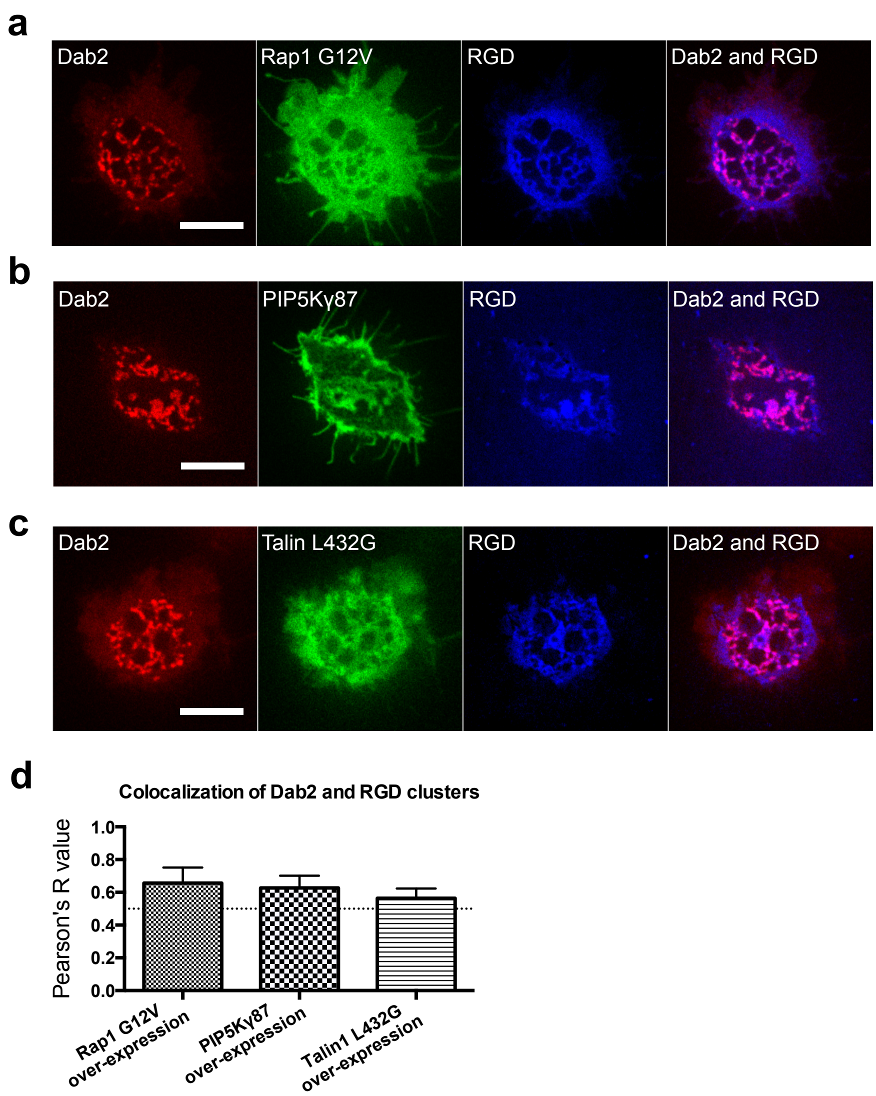

Supplementary Figure 6. Dab2 remains recruited to RGD/integrin-beta3 clusters even with mutants to pro-activate integrins. (a-c) Overexpression of GFP-Rap1 V12, GFP-PIP5Kγ87, and calpain-cleavage resistant mutant GFP-talin-L432G do not block Dab2-mCherry recruitment at RGD clusters. (d) Pearson's correlation analysis of Dab2 and RGD clusters, with overexpression of Rap1 V12, PIP5Kγ87, and talin-L432G. All data are the mean of three independent experiments (n > 20 in each condition). Error bars represent s.d., and all samples represent biological replicates. Detailed statistical results are available in Supplementary Table 1. Scale bar represents 10μm.

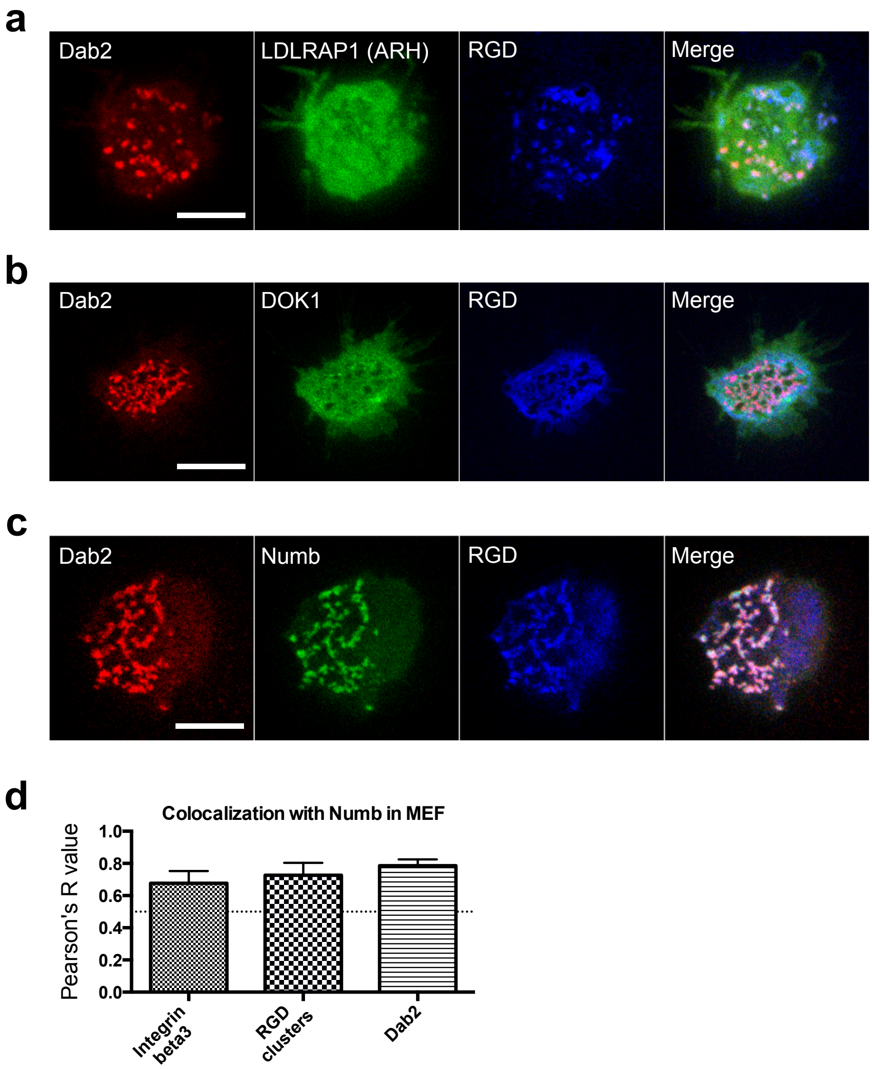

Supplementary Figure 7. PTB domain containing protein Numb is recruited at RGD clusters. (a) The localization of PTB-containing adaptor proteins LDLRAP1-GFP at RGD clusters is low and limited. (b) DOK1-GFP is not enriched at Dab2-bound RGD clusters. (c) PTB-containing adaptor protein Numb-GFP is colocalized with Dab2-mCherry and RGD clusters. (d) Pearson's correlation analysis of Numb/integrin-beta3, Numb/RGD, and Numb/Dab2. All data are the mean of three independent experiments (n > 20 in each condition). Error bars represent s.d., and all samples represent biological replicates. Detailed statistical results are available in Supplementary Table 1. Scale bar represents 10μm.

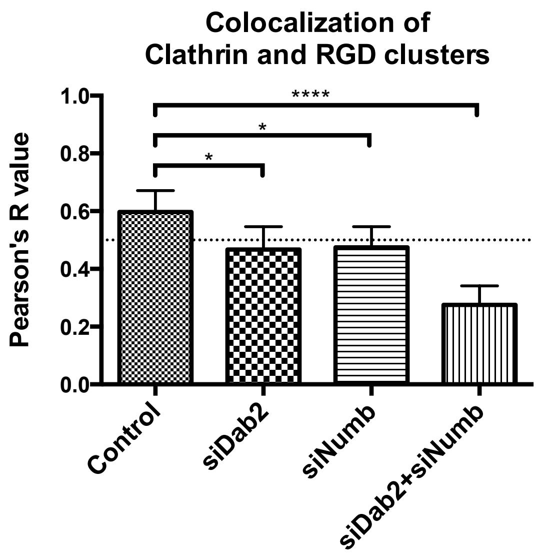

Supplementary Figure 8. Colocalization analysis of clathrin and RGD. Single siRNA knockdown of Dab2 and Numb causes a small decrease of colocalization of clathrin and RGD. Double knockdown of Dab2 and Numb results in further decline in colocalization. The knockdown efficiency is found to be 70-90% (Fig. 5c and 5d). All data are the mean of three independent experiments (n > 20 in each condition). P values by unpaired Kruskal– Wallis test are: **** P < 0.0001, and * P < 0.05. Error bars represent s.d., and all samples represent biological replicates. Detailed statistical results are available in Supplementary Table 1.

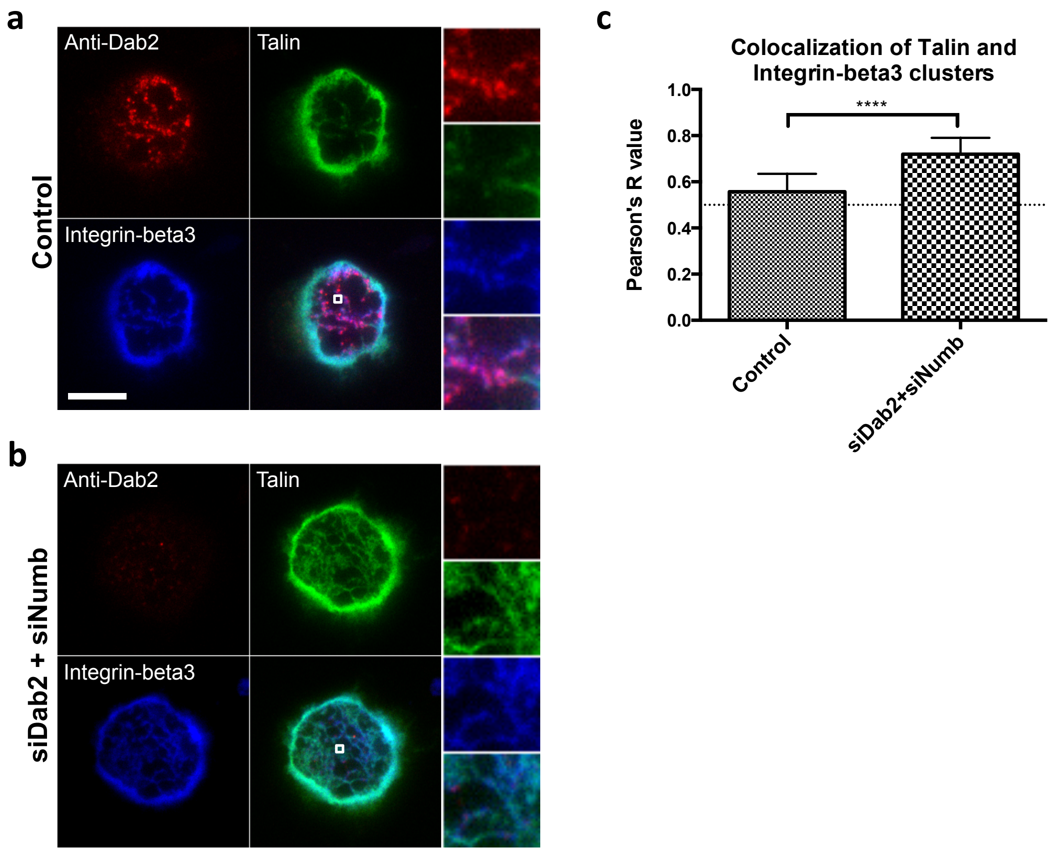

Supplementary Figure 9. Increased colocalization of talin and integrin-beta3 in the Dab2/Numb knockdown cells. (a) Anti-Dab2 and GFP-talin are spatially separated at integrin-beta3-BFP2 clusters. Talin is enriched at integrin-beta3 clusters in the periphery of the adhesion zone, but absent at integrin-beta3 clusters in the center of the adhesion zone. (b) Double knockdown of Dab2 and Numb causes the increased colocalization of GTP-talin and integrin-beta3-BFP2. (c) Pearson's correlation analysis of talin and integrin-beta3. All data are the mean of three independent experiments (n > 20 in each condition). Error bars represent s.d., and all samples represent biological replicates. Detailed statistical results are available in Supplementary Table 1. Scale bar 10 μm.

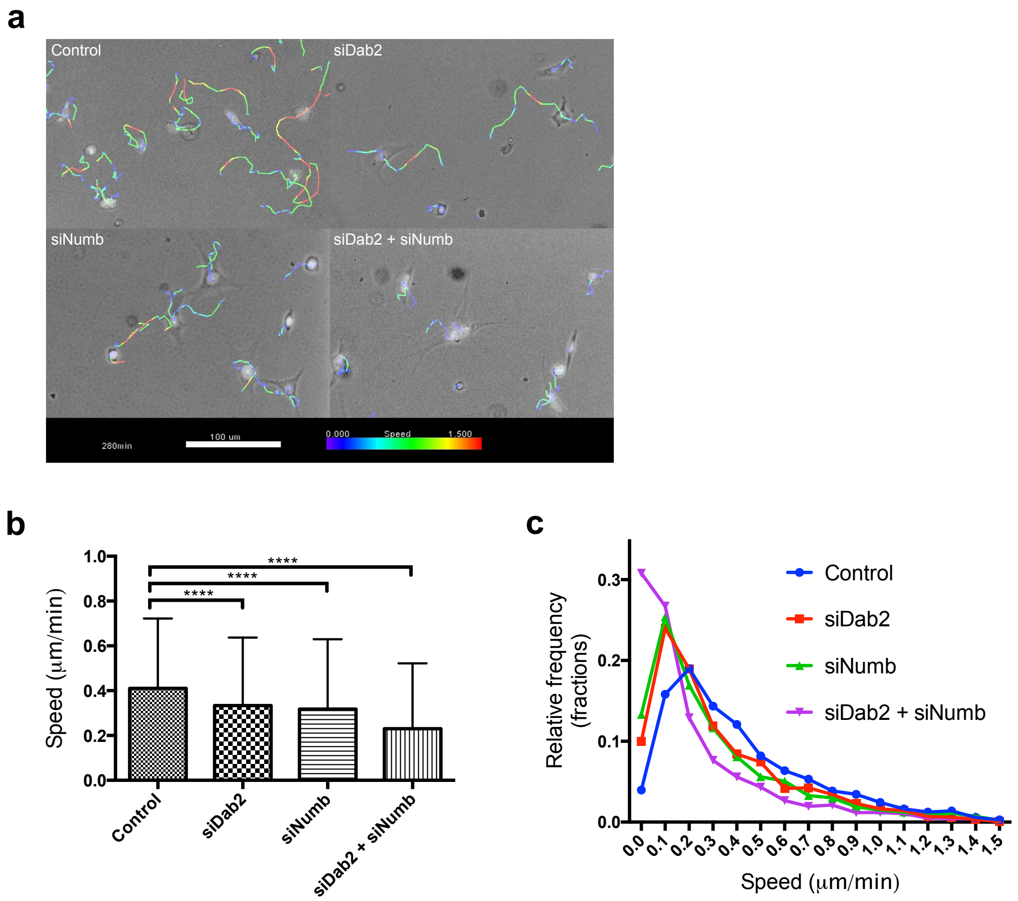

Supplementary Figure 10. Knockdown of Dab2 and Numb reduces cell migration speed. (a) Migration tracks of control, siDab2, siNumb, and siDab2+siNumb cells on compliant fibronectin-coated PDMS gel (3kPa). Color-coded tracks represent migration speed within 10hrs of observation. Also see Supplementary Movie 5. Scale bar 100μm. (b and c) Single knockdown of Dab2 or Numb causes a decrease in cell migration speed, and dual knockdown of both Dab2 and Numb causes yet further decrease in migration speed. P values by unpaired Kruskal–Wallis test are: **** $P < 0.0001$. All data are individual cell migration speed ($n > 2000$ in more than 30 cells in each condition) in two independent experiments. Error bars represent s.d., and all samples represent biological replicates. Detailed statistical results are available in Supplementary Table 1.

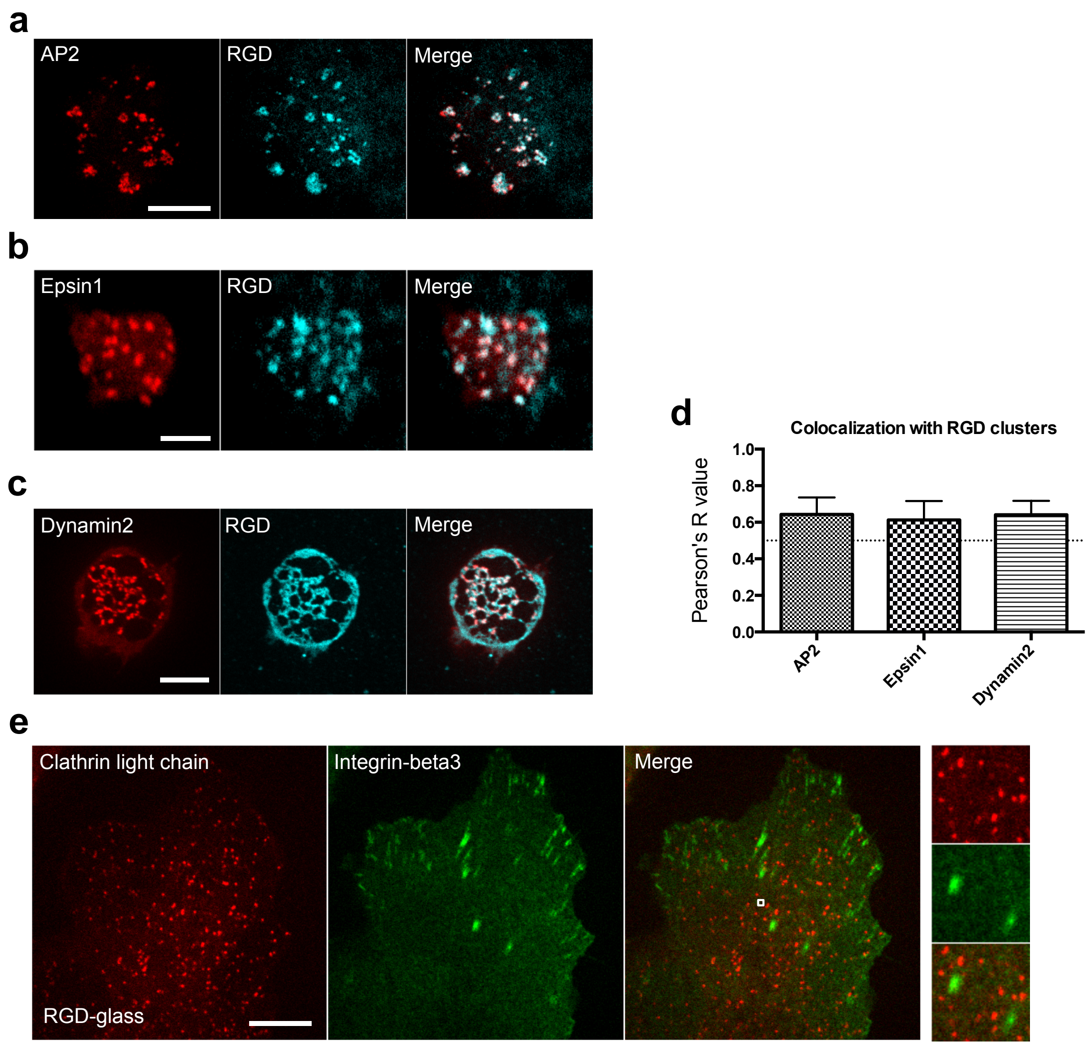

Supplementary Figure 11. Clathrin-mediated endocytic machineries are recruited at RGD clusters. (a-c) AP2-mCherry, epsin1-CFP, and mCherry-dynamin2 in REF52 fibroblasts localize with RGD clusters. (d) Pearson's correlation analysis of RGD/AP2, RGD/epsin1, and RGD/dynamin2. (e) mCherry-clathrin light chain was absent at integrin-beta3-GFP adhesion plaques when the MEF adhered on RGD-glass. All data are the mean of three independent experiments (n > 20 in each condition). Error bars represent s.d., and all samples represent biological replicates. Detailed statistical test results are available in Supplementary Table 1. Scale bar represents 10μm.

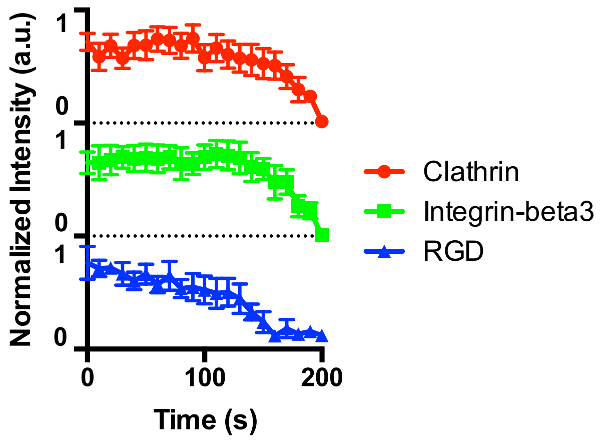

Supplementary Figure 12. Synchronized intensity decreases in integrin-beta3, clathrin, and RGD. In the case of endocytic active puncta, the intensity profiles of integrin-beta3, clathrin, and RGD decrease together. Also see Fig. 6b. Error bars represent s.e.m. (n=6).

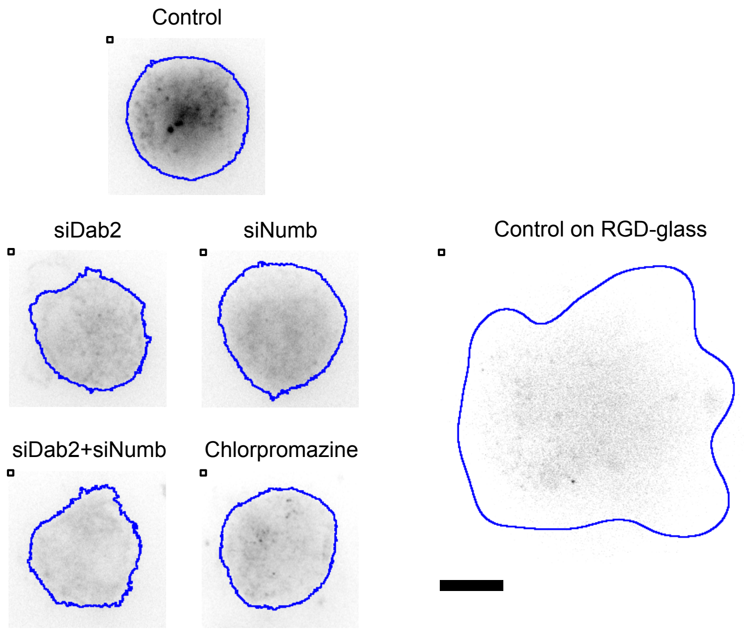

Supplementary Figure 13. Endocytosis of RGD ligands. Images represent inverted maximum projection of the confocal planes (1-4 μm above the substrate) of endocytosed RGD in control, siDab2, siNumb, siDab2+siNumb, and chlorpromazine treated cells on RGD-membranes, as well as control cells on RGD-coated glass. Also see Fig. 7b. Scale bar 10 μm.

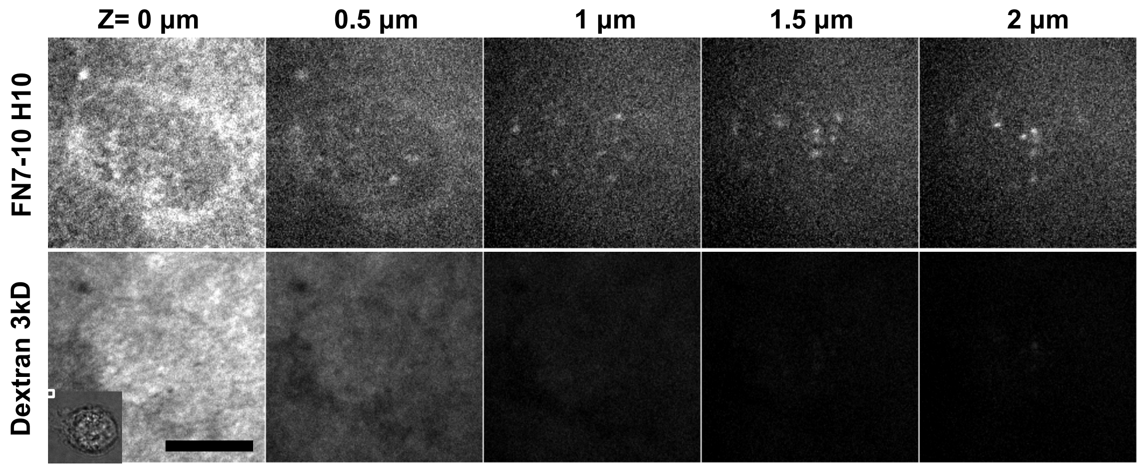

Supplementary Figure 14. FN7-10 ligands on supported membranes are selectively endocytosed. Fibronectin repeat III 7th to 10th domain monomer with ten histidines (FN7-10 H10) contains the RGD motif for integrin activation and is labeled by CF680R. When CF680R-FN7-10 H10 and micro-ruby dextran (3kD, with biotin and tetramethylrhodamine labels) are both presented on supported membranes, endocytosis of FN7-10, not dextran is observed. Scale bar represents 10μm.

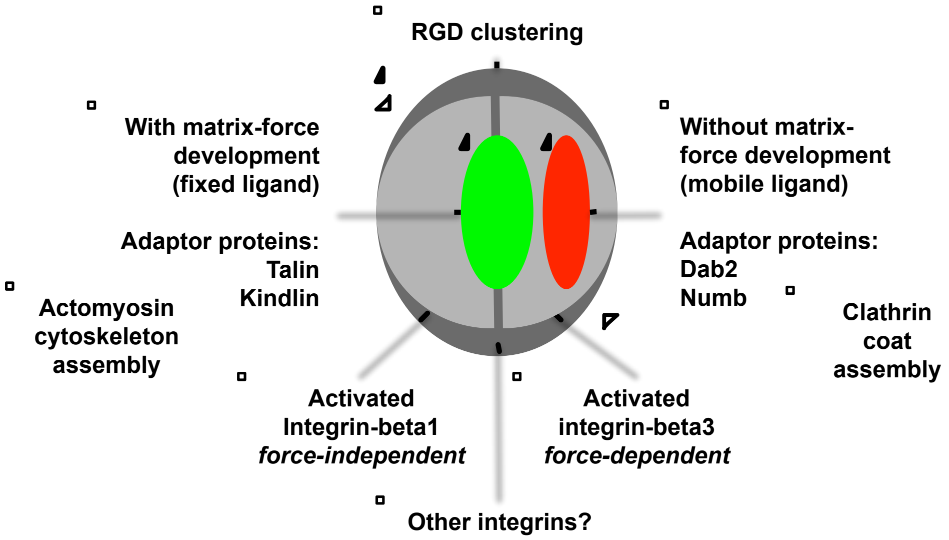

Supplementary Figure 15. Summary of the proposed model. Matrix-force development mediates adaptor protein binding to RGD-integrin-beta3 clusters.

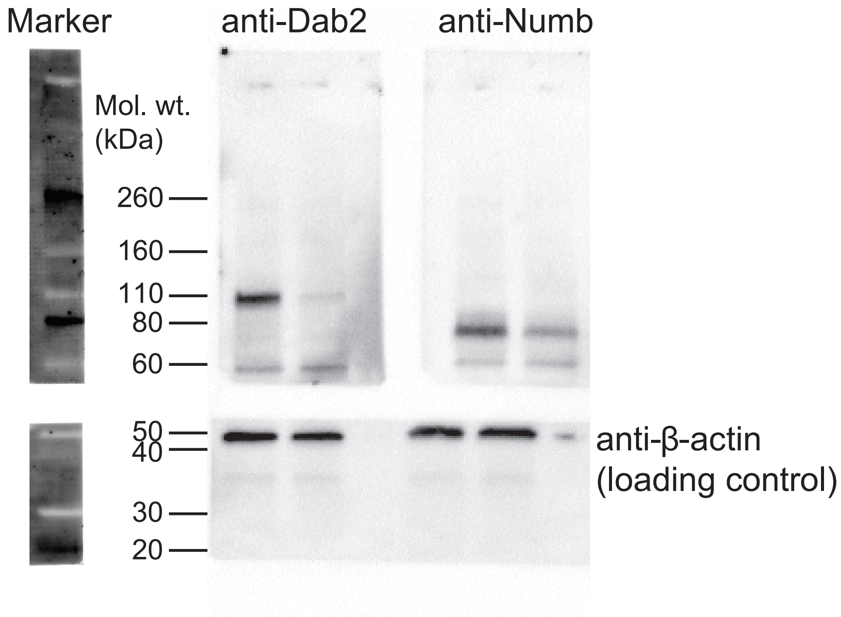

Supplementary Figure 16. Full western blots of the siDab2 and siNumb knockdown in MEF. The blots are stained and then recorded by a CCD camera.

Supplementary Table 1: Detailed statistical test results

Figure 1d

	Beta3 (LIBS2)	RGD	Beta1 (9EG7)
Cell numbers	28	25	23
Mean	0.5911	0.6248	0.15
SD	0.05711	0.0465	0.105
P values		> 0.05	< 0.0001

Figure 1e

	Beta3 (LIBS2)	Beta3-GFP
Cell numbers	31	24
Mean	0.7558	0.6956
SD	0.04931	0.07939
P values		> 0.05

Figure 1j

	Mobile zone	Fixed zone
Cell numbers	24	30
Mean	0.5888	0.283
SD	0.06313	0.1168
P values		< 0.0001

Figure 2b

	RGD membrane	RGD glass
Cell numbers	46	38
Mean	0.4694	0.7674
SD	0.09201	0.104
P values		< 0.0001

Figure 2f

	MEF WT	RhoA Q63L	MRLC T18S19D
Cell numbers	44	36	25
Mean	0.6291	0.2221	0.2912
SD	0.08954	0.1361	0.1556
P values		< 0.0001	< 0.0001

Figure 3b

	β3	β1	β3-Y772F	β3-Y784F	β3 + PP2
Cell numbers	21	24	38	42	36
Mean	0.5648	0.1625	0.08254	0.3076	0.092
SD	0.07821	0.1012	0.1753	0.2112	0.1582
P values		< 0.0001	< 0.0001	< 0.001	< 0.0001

Figure 4i

	Talin1	Kindlin1	Tensin1	FilaminA
Cell numbers	22	24	27	22
Mean	-0.02846	0.1125	0.03067	-0.032
SD	0.1119	0.1166	0.08242	0.1329

Figure 5b

	Dab2	Integrin beta3	RGD clusters
Cell numbers	23	27	26
Mean	0.776	0.6756	0.67
SD	0.09477	0.07939	0.06988

Figure 6c, 6d

	Active	Total	Ratio
Cell numbers	6	6	6
Mean	27	160.2	0.1664
SD	11.56	42.64	0.03809

Figure 7a

	α5β1 + αvβ3	α5β1
Cell numbers	23	22
Mean	3.722	0.6266
SD	0.7404	0.1444
P values		< 0.0001

Figure 7b

	Wildtype	siDab2	siNumb	siDab2+siNumb	Chlorpromazine	Wildtype of glass
Cell numbers	37	33	29	32	36	43
Mean	3467	1804	2224	1747	1482	901.7
SD	506.4	439.5	359.4	387.9	376.1	343.4
P values		< 0.0001	< 0.01	< 0.0001	< 0.0001	< 0.0001

Continue on the next page

Supplementary Table 1: Detailed statistical test results

Supplementary Figure 1b

	RGD	RGE	RAD
Cell numbers	387	401	421
Mean	77.86	14.56	18.27
SD	6.913	4.128	6.253
P values		< 0.001	< 0.01

Supplementary Figure 2d

	Beta3	CA-LRP6
Cell numbers	25	22
Mean	0.61	0.2371
SD	0.07141	0.06775
P values		< 0.001

Supplementary Figure 6d

	Rap1 G12V	PIP5Kγ87	Talin1 L432G
Cell numbers	28	21	22
Mean	0.6564	0.6271	0.5645
SD	0.0954	0.07477	0.05922

Supplementary Figure 7d

	Beta3	RGD	Dab2
Cell numbers	21	29	26
Mean	0.6763	0.7265	0.7856
SD	0.07581	0.07693	0.03999

Supplementary Figure 8

	Control	siDab2	siNumb	siDab2+siNumb
Cell numbers	26	26	25	29
Mean	0.5969	0.4669	0.4747	0.2758
SD	0.07418	0.07905	0.0712	0.06552
P values		< 0.05	< 0.05	< 0.0001

Supplementary Figure 9c

	Control	siDab2+siNumb
Cell numbers	25	22
Mean	0.5571	0.7196
SD	0.07776	0.0708
P values		< 0.0001

Supplementary Figure 10b

	Control	siDab2	siNumb	siDab2+siNumb
Speed dataset	2389	4899	4304	4713
Mean	0.410549	0.333374	0.318267	0.230431
SD	0.311499	0.303535	0.311474	0.291741
P values		< 0.0001	< 0.0001	< 0.0001

Supplementary Figure 11d

	AP2	Epsin1	Dynamin2
Cell numbers	30	23	24
Mean	0.6433	0.6127	0.6408
SD	0.09292	0.104	0.07669
